# Shifts in the Fecal Microbial Community of *Cystoisospora suis* Infected Piglets in Response to Toltrazuril

**DOI:** 10.3389/fmicb.2020.00983

**Published:** 2020-05-19

**Authors:** Aruna Shrestha, Barbara U. Metzler-Zebeli, Hamadi Karembe, Daniel Sperling, Simone Koger, Anja Joachim

**Affiliations:** ^1^Department of Pathobiology, Institute of Parasitology, University of Veterinary Medicine Vienna, Vienna, Austria; ^2^Unit Nutritional Physiology, Department of Biomedical Sciences, Institute of Physiology, Pathophysiology and Biophysics, University of Veterinary Medicine Vienna, Vienna, Austria; ^3^Ceva Santé Animale, Libourne, France; ^4^Department of Farm Animals and Veterinary Public Health, Institute of Animal Nutrition and Functional Plant Compounds, University of Veterinary Medicine Vienna,, Vienna, Austria

**Keywords:** piglet, microbiota, gut health, 16S rRNA, diarrhea

## Abstract

The protozoan parasite *Cystoisospora suis* causes diarrhea and reduced weight gain in suckling piglets. Infections occur in the first days of life; it is transient but can lead to dysbiosis, exacerbating disease and increasing mortality. Cystoisosporosis is effectively controlled by toltrazuril treatment; however, alterations of the gut microbial composition upon infection and treatment have not been investigated. This study evaluated the development of fecal microbiota of *C. suis* infected piglets in response to treatment with toltrazuril. Thirty-eight conventional piglets were infected with *C. suis* on the first day of life (dol 1). Twenty-six of them received either parenteral or oral toltrazuril 2 days later. Fecal samples were collected pre- and post-weaning (dol 1–15 and 31–38) for microbiota analysis using 16S rRNA amplicon sequencing and during dol 5–18 to determine fecal consistency and parasite excretion. All control animals shed parasites at least once and the majority developed diarrhea, while toltrazuril-treated piglets did not excrete parasites and only had low levels of diarrhea. Age-related shifts in the fecal microbiota composition and increase in diversity and species richness were seen until after weaning. Parasite infection disrupted bacterial maturation 2 weeks after infection. Irrespective of the route of administration, fecal communities of piglets in the treated groups clustered separately and were more diverse compared to that of control piglets during the acute phase of infection on dol 11. Control piglet feces showed higher levels of *Fusobacteriaceae* and *Veillonellaceae*, while *Ruminococcaceae*, *Lachnospiraceae*, *S24-7*, *Clostridiaceae*, and *Erysipelotrichaceae* were more abundant in feces of treated piglets on dol 11. Thereafter, treatment-related effects on the microbial communities were small and mainly detectable on dol 34 (5 days post-weaning), potentially indicating that the oral toltrazuril treatment might have had long-term effects on host physiological responses post-weaning. Irrespective of the administration route, toltrazuril prevented *C. suis*-related dysbiosis and maintained species richness and diversity on dol 11. In addition to cystoisosporosis prevention, toltrazuril seems to contribute to the stabilization of the gut microbial development during the suckling phase and thus may reduce the need for antibiotics to control infections with secondary bacterial enteropathogens in *C. suis-*infected suckling piglets.

## Introduction

The genus *Cystoisospora* [formerly known as *Isospora;* (Barta et al., 2005)] encompasses important enteric protozoan pathogens of humans, swine, dogs and cats (Lindsay et al., 1997). *Cystoisospora suis* (syn. *Isospora suis*) causes one of the most prevalent and economically important diarrheic diseases of suckling piglets, porcine cystoisosporosis, with worldwide distribution (Joachim and Shrestha, 2020). It entirely develops in one host. After ingestion of environmentally persistent stages, the oocysts, a complex development is initiated during which invasive stages are released and infect epithelial cells of the small intestine to reproduce asexually within an intracellular vacuole. After this phase of rapid multiplication (merogony), the parasites differentiate into sexual stages, whereupon cellular fusion and formation of a zygote occur. The zygote forms the oocyst wall and is excreted in an immature state. In the environment, maturation via meiosis occurs and the life cycle is completed. The rapid development of *C. suis* (5 to 5 days) ensures a fast spread within and between litters of newborn piglets [for review see (Joachim and Shrestha, 2020)].

Merogony occurs multiple times during development, resulting in massive injury of the intestinal lining, such as desquamation of the epithelium and atrophy, fusion and necrosis of the villi, as well as crypt hyperplasia. Pathological alterations due to cystoisosporosis persist for a considerable time after completion of parasite development (up to 2 weeks after infection) until the epithelial integrity is fully restored (Joachim and Shrestha, 2020). Impaired intestinal homeostasis and slow regeneration of the intestinal epithelium in infected piglets lead to significant reductions in body weight gain, most likely due to reduced nutrient absorption (Mundt et al., 2003, 2006; Joachim and Mundt, 2011). The disease is effectively controlled by oral or parenteral administration of a single dose of the triazinone toltrazuril during the developmental phase of *C. suis* in the host (Mundt et al., 2006; Joachim et al., 2018c, 2019). The exact mode of action of toltrazuril is not known but it has been suggested that toltrazuril interferes with nuclear division and mitochondrial activity in coccidian parasites (Harder and Haberkorn, 1989). Despite its action against these pathogenic intestinal protozoa, conclusive information about the impact of toltrazuril administration on the gut microbiota in pigs is missing.

Although *C. suis* is a primary pathogen (Harleman and Meyer, 1984, 1985), mortality due to uncomplicated cystoisosporosis is usually low (Stuart et al., 1980; Meyer et al., 1999). However, synergistic actions of *C. suis* with other enteric bacterial and viral pathogens can exacerbate the disease, thereby increasing mortality (Vítovec et al., 1991; Driesen et al., 1993; Mengel et al., 2012). For *Clostridium perfringens* type A (*Cp*A) increased growth and adhesion in the presence of *C. suis* could be demonstrated in experimental infections with *C. suis*, and anticoccidial treatment with toltrazuril also prevented *Cp*A overgrowth and *Cp*A-induced necrotic enteritis (Mengel et al., 2012), indicating a synergistic relationship between *C. suis* and *Cp*A. On the other hand, probiotic bacteria had no appreciable influence on the course of *C. suis* infection despite a beneficial effect in healthy piglets (Unterweger et al., 2018). This indicates that *C. suis* induces intestinal microbiota disruption in pre-weaned piglets, not *vice versa*.

The intestinal colonization of the neonatal porcine gut starts right after birth, and the gut microbial composition and ecological succession in early life are influenced by a number of complex internal and external factors (Guevarra et al., 2019). *C. suis* infections displays a strong age resistance, and piglets older than 3 weeks are rarely affected (Lindsay et al., 1985; Koudela and Kučerová, 1999; Worliczek et al., 2009). The consequences of infection are therefore most serious during the development of the gut microbiota in an early stage of life. However, apart from selected bacterial enteropathogens like *Cp*A, very little is known about the influence of *C. suis* on the gut microbiota development. Recent evidence suggests that not just specific pathogens but gut microbial shifts contribute to the development of diarrhea in weaned piglets (Dou et al., 2017), and this may be promoted by *C. suis* infections.

We hypothesized that treatment with toltrazuril rectifies disruption of the early gut microbiota development induced by *C. suis* infection. The rapid and direct transmission of *C. suis* between piglets does not allow inclusion of non-infected littermates. In order to inhibit the pathological and clinical effects of *C. suis* and to restore gut health, a part of the animals was treated with the coccidiocidal drug toltrazuril either parenterally or orally during the prepatent phase of infection on day of life (dol) 2. The effect of toltrazuril treatments on the development of the gut microbiota of infected piglets was central to this investigation.

## Materials and Methods

### Sample Collection and Processing

Parasite propagation and study design, including experimental infection and treatment, were previously described (Joachim et al., 2019) with additional fecal samplings for microbiota analysis. In brief, 38 conventionally raised piglets (Landrace × Large White) from three crossbred sows were randomly allocated to three treatment groups. All piglets were orally inoculated with 1,000 sporulated oocysts of *C. suis* in 1 ml of water on the first dol. Each piglet in the parenteral toltrazuril group (*n* = 13) received a fixed dose of 45 mg of toltrazuril + 200 mg of iron as gleptoferron (1.5 ml; Forceris^®^, Ceva Santé Animale, France) on the second day of life by intramuscular injection and piglets in the group oral toltrazuril (*n* = 13) received 20 mg/kg body weight of toltrazuril (Baycox^®^, Bayer, Germany) *per os* on the fourth day of life. All piglets in the group oral toltrazuril and the control group (*n* = 11) received 200 mg of parenteral iron (Uniferon^®^ 200, Virbac, Holbaek, Denmark) per piglet on the second day of life for the prevention of iron deficiency anemia. The piglets received milk from the sow followed by piglet pre-starter (Garant Tiernahrung GmbH, Pöchlarn, Austria; crude protein: 17.5%, fat: 9.0%; and crude fiber: 2.5%) *ad libitum* from the second week of life and had free access to the sow’s feed (Garant-Tiernahrung GmbH, Pöchlarn, Austria; crude protein: 15%, fat: 4.0%; and crude fiber: 5%) as well as straw which was used as bedding material. Piglets were weaned on dol 29 and moved to separate boxes with straw bedding where they received piglet starter diet (Garant-Tiernahrung GmbH, Pöchlarn, Austria; crude protein: 17%, fat: 3.5%; and crude fiber: 3.5%) and water *ad libitum*.

Individual fecal samples were collected daily from dol 5 to dol 18 to investigate progression of infection and development of disease, and to evaluate the efficacy of treatment. Immediately after collection, fecal samples were evaluated for consistency (normal or diarrheic) and screened qualitatively for the presence of oocysts by autofluorescence, followed by quantitation by a modified McMaster technique (Joachim et al., 2018b). All piglets were weighed at birth and then weekly on dol 8, 15, 22, and 29 to determine body weight gain.

For analysis of the fecal bacterial microbiota, additional fecal samples were collected directly from the rectum of all piglets in each group on dol 1, 3, 5, 11, 15 (before weaning) and dol 31, 34 and 38 (after weaning on dol 29). Fecal consistency was scored immediately after sampling as meconium (dol 1), normal or diarrheic. All fecal samples were stored at −80°C until further processing. Only piglets with a complete set of fecal samples were selected for 16S rRNA gene sequencing analysis.

### DNA Extraction and Amplification

Genomic DNA was extracted from fecal samples as described previously (Metzler-Zebeli et al., 2019). Then, the V3-V4 region of the 16S ribosomal RNA was amplified and sequenced (Klinsoda et al., 2019). Amplicon sequencing was performed on an Illumina MiSeq sequencing platform by a commercial provider (Microsynth, Balgach, Switzerland). This included the 16S rRNA PCRs, library preparation, and sequencing. The primers 341F-ill (5′-CCTACGGGNGGCWGCAG-3′) and 802R-ill (5′-GACTACHVGGGTATCTAATCC-3′) were used to target the V3-V4 hypervariable regions of the bacterial 16S rRNA gene, which generate an amplicon of approximately 460 bp. The 16S rRNA PCRs were performed using the KAPA HiFi HotStart PCR Kit (Roche, Baden, Switzerland). The Nextera XT DNA Sample Preparation Kit (Illumina) was used for preparation of libraries by ligating sequencing adapters and indices onto the purified PCR products. After library normalization, the equimolar quantities of each library were pooled and sequenced on an Illumina MiSeq sequencing v2 platform using a paired-end protocol. Subsequently, reads were demultiplexed and adapter sequences were removed using cutadapt^1^.

Absolute quantification of total bacteria in fecal samples was performed on a Stratagene Mx3000P qPCR system (Agilent Technologies) using previously published primer set (Muyzer et al., 1993) and amplification conditions (Klinsoda et al., 2019). Reactions including samples, negative controls and the reverse transcription controls (RT minus) were run in duplicate on each plate. Standard curves were prepared from 10-fold serial dilutions (10^7^ to 10^3^ molecules/μl) of the purified and quantified PCR products using genomic DNA from pig feces of the present study as described (Klinsoda et al., 2019).

### Bioinformatic Analysis and Statistical Analysis

Sequencing data were analyzed using the DADA2 package (version 1.12.1) in R studio (version 1.0.136). After inspecting the quality profiles of the forward and reverse reads separately, the first 10 nucleotides for each read were trimmed and the total length of reads were truncated to 220 nucleotides to account for the large decrease in quality score observed thereafter. Moreover, all reads containing any ambiguities were removed as were reads exceeding the probabilistic estimated error of two nucleotides. After de-replication of the filtered data, error rates were estimated and amplicon sequence variants (ASVs) were inferred (Callahan et al., 2016). The DADA2 method thereby infers ASVs exactly without imposing arbitrary thresholds, which allows resolving ASVs that differ by as little as one nucleotide (Callahan et al., 2016). Afterward, the inferred forward and reverse sequences were merged, whereby paired sequences that did not perfectly match were removed to control against residual errors, and a sequence table was built. After chimera removal using the removeBimeraDenovo() function, taxonomy was assigned using the Greengenes database (version 13_8) with a dissimilarity threshold of 3%.

Diversity (α and β) and community composition analyses were performed using the phyloseq R package and the vegan R package (Oksanen et al., 2018). For α-diversity (Shannon, Simpson, Chao1) analysis, the samples were rarified to an equal library size using the “rarefy_even_depth()” function in phyloseq, thereby removing 844 ASVs. The adonis2 function in the vegan R package was used to statistically assess dissimilarity matrices (Bray-Curtis) derived from the microbiota data at genera level (relative abundance >0.05%). Clustering of fecal samples from the treatment groups and dol were visualized in two-dimensional non-metric multidimensional scaling (NMDS) ordination plots obtained with the “metaMDS” function in the vegan R package (Oksanen et al., 2018). To identify the most discriminant genera which have influenced α-diversity, growth performance, fecal score and oocyst excretion in feces, multigroup supervised DIABLO N-integration networking was performed using the mixOmics R package (version 6.3.2) (Rohart et al., 2017) as described previously (Klinsoda et al., 2019; Metzler-Zebeli et al., 2019). Sparse partial least square regression enabled the discrimination of genera across treatment groups with the lowest possible error rate selecting 10 genera, which were associated with α-diversity, growth performance, fecal score and oocyst excretion in feces. Only the strongest associations were projected using relevance networking and the “network” function in mixOmics. Additionally, horizontal sparse partial least square discriminant analysis (sPLS-DA) using the “block.splsda” function was applied to identify the most discriminant genera (*n* = 10) across treatments which were presented in loading plots.

The raw sequence counts from the taxa tables were collapsed and compositionally normalized such that each sample summed to 1. The relative abundances at the respective taxonomic rank were analyzed. All variables were tested for normal distribution by the Shapiro-Wilk test with the UNIVARIATE procedure in SAS (Version 9.4, SAS Inst. Inc., Cary, NC, United States). Repeated measures were used to assess differences in the total bacterial 16S rRNA gene copies, species richness and α-diversity indices among dol using the MIXED procedure in SAS. To compare differences between treatments and dol, data for α-diversity, predominant bacterial phyla and families were subjected to ANOVA using the MIXED procedure in SAS. The model included the fixed effects of treatment, dol and their 2-way-interaction and litter and fecal consistency as random effect. Pig was the experimental unit. The degrees of freedom were approximated by the Kenward-Rogers method (ddfm = kr). The means were reported as least-squares means ± standard error of the mean (SEM). The differences were considered significant if *P* < 0.05.

## Results

### Clinical and Parasitological Outcome of Experimental Infections

Oocyst excretion is a primary parameter to estimate the intensity and clinical presentation of *C. suis* infection (Joachim et al., 2018a). Detailed results of the clinical and parasitological outcome of experimental infections of the involved animals have been published previously (Joachim et al., 2019). Briefly, three groups of piglets (*n* = 11–13 animals/group) were used. All piglets were infected with *C. suis* on the first day of life (dol). Two groups were treated with toltrazuril, one with an oral suspension on the fourth dol, the other one parenterally on the second dol. In the untreated control group, all piglets excreted oocysts at least once and the prevalence of McMaster countable excretion reached a first peak on dol 8, and a second one on the dol 13 (Supplementary Figure S1). Oocyst excretion started from dol 7 and lasted until the end of the sampling period. None of the piglets from the treated groups excreted oocysts detectable in autofluorescence or the McMaster technique. Diarrhea developed as early as dol 7, with an average duration of 3.6 days per piglet. In contrast, none of the treated piglets had diarrhea during the acute phase of parasite development, except for one piglet in the parenteral group, which showed diarrhea for 5 days. The onset of diarrhea in this piglet was on dol 5, 2 days earlier than that of the control group. After weaning, none of the piglets showed diarrhea. Body weights were not significantly different on dol 1 and 8. However, mean body weights were significantly lower (*P* < 0.05) in the control group on dol 15, resulting in lower daily body weight gain (447 g) during the acute phase of infection compared to that of the treated groups (1486 and 1447 g) (Supplementary Table S1).

### Development of the Fecal Bacterial Microbiota

Between 32 and 35 samples (= pigs) were available for 16S rRNA gene sequencing analysis for each sampling day (*n*_total_ = 277). Of note, on the first dol, 12 of the samples were meconium. After quality control and chimera check, a total of 7,182,975 sequencing reads with a mean of 25,931 reads per sample were obtained from 16S rRNA amplicon sequencing (V3-V4) for the 277 fecal samples (mean read length: 418 bp). The samples comprised 24 phyla, which contained a total of 151 families, 339 genera and 7055 amplicon sequence variants (ASVs). Across all time points and treatment groups, the most relatively abundant phyla were *Firmicutes* (46.6%), *Bacteroidetes* (26.9%), *Fusobacteria* (11.3%), and *Proteobacteria* (11.1%). At the family level, *Fusobacteriaceae* (11.3%), *Prevotellaceae* (10.5%), *Lachnospiraceae* (10.2%), *Ruminococcaceae* (9.6%), and *Bacteroidaceae* (9.0%) were dominant.

**FIGURE 1:**
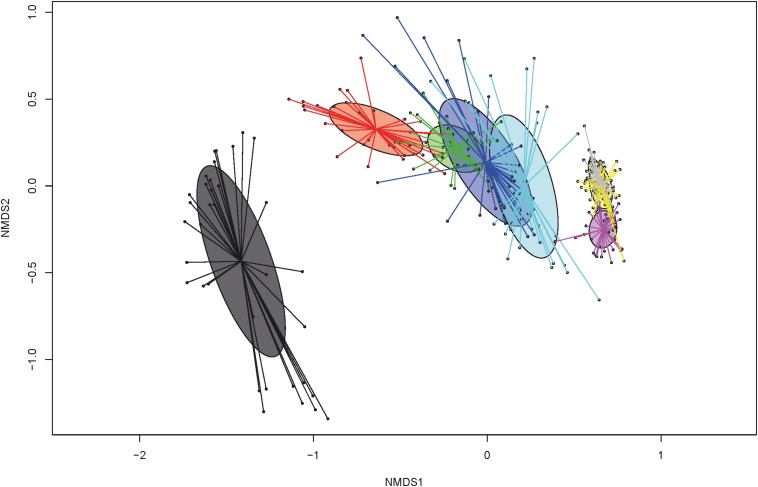
Non-metric multidimensional scaling (NMDS) plot of pairwise Bray-Curtis dissimilarities among bacterial communities over time at days of life: 1 (dark gray), 3 (red), 5 (green), 11 (dark blue), 15 (light blue), 31 (pink), 34 (yellow), and 38 (light gray).

**FIGURE 2:**
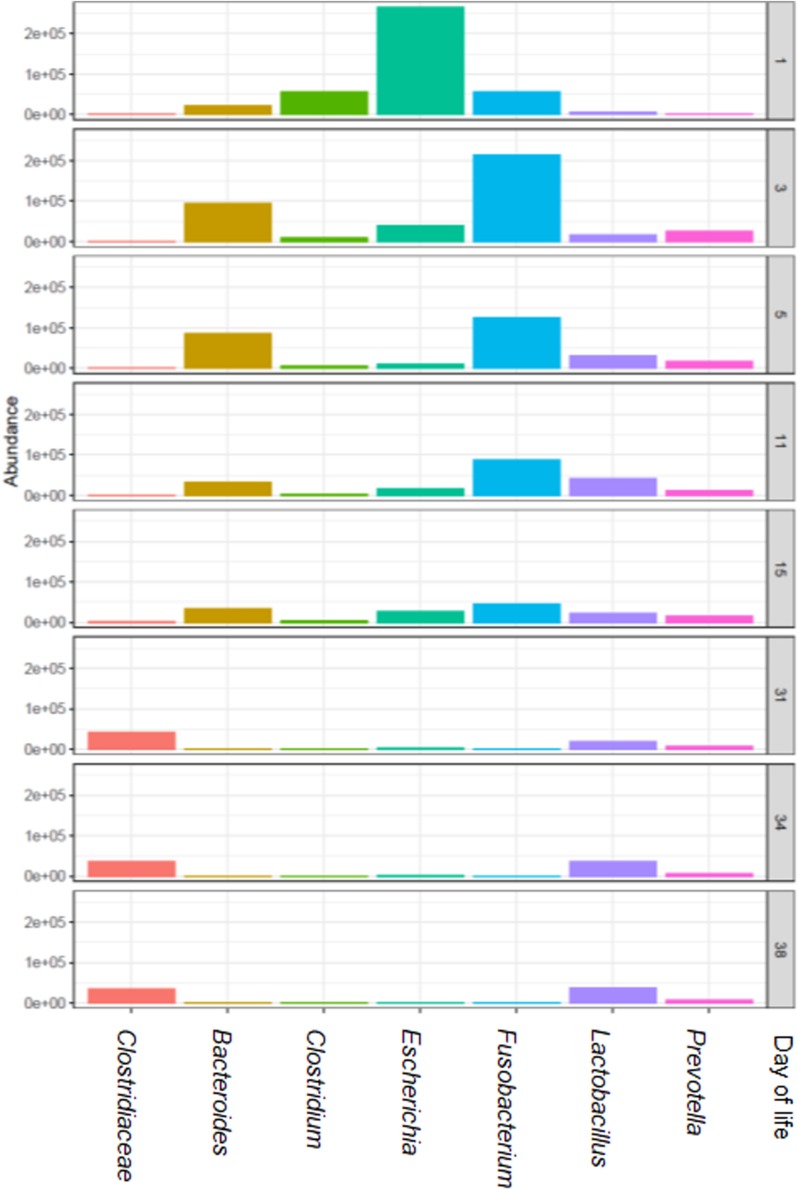
The most relatively abundant genera (hit counts) in feces in relation to piglet age (day of life 1, 3, 5, 11, 15, 31, 34, and 38).

When the total number of 16S rRNA gene copies was compared by dol for all groups, the numbers increased from the first dol and decreased sharply on dol 15 during the suckling phase. Post-weaning, total 16S rRNA gene copies increased again to a level comparable to dol 11. Bacterial communities evolved over time with three main clusters (Bray-Curtis dissimilarities), which correspond to the first dol, the suckling period (dol 3 to 15) and the post-weaning period (dol 31 to 38) (Figure 1). Concerning bacterial diversity, species richness and diversity increased with age. Weaning (on dol 29) noticeably increased species richness and diversity (Shannon index) (Figure 1). The relative abundance of the most abundant genera changed rapidly from dol 1 with a dominance of *Escherichia* to a predominance of *Fusobacterium* from dol 3 to 15 during the suckling phase, while after weaning an unclassified *Clostridiaceae* genus, *Lactobacillus* and *Prevotella* were the most abundant genera (Figure 2).

### Effects of Toltrazuril Treatment on the Fecal Bacterial Microbiota

**FIGURE 3:**
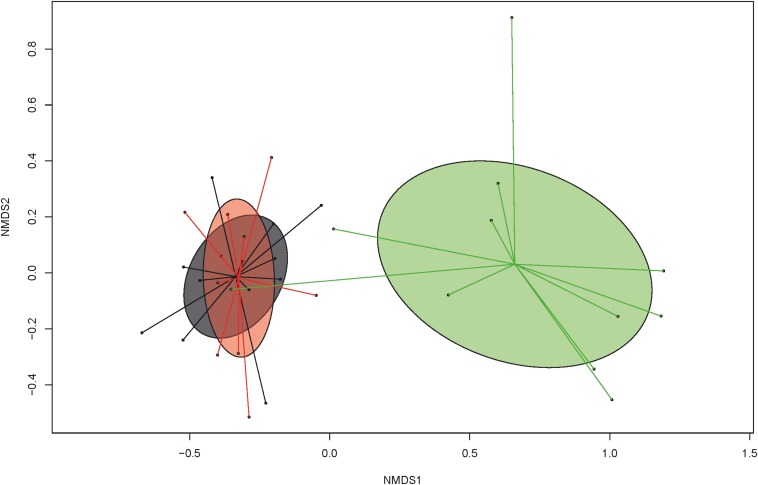
Non-metric multidimensional scaling (NMDS) plot of pairwise Bray-Curtis dissimilarities among bacterial communities at the genus level in feces of suckling and weaned piglets (>0.01% relative abundance) demonstrating treatment effect (green: group C = untreated control), compared to treated groups A (gray = parenteral toltrazuril) and B (red = oral toltrazuril) on day of life 11 (stress value: 0.1273; stress value = approximation for the strength of the pairwise dissimilarities and are in relation to the axes dimensions of the graph).

Toltrazuril treatment modified the diversity and composition of the fecal bacterial communities. Species richness and evenness (Simpson and Shannon) were reduced (*P* < 0.05) in the control group compared to the two treatment groups on dol 11 (species richness, Simpson and Shannon indexes; Table 1). Likewise, Bray-Curtis-derived dissimilarities showed distinct clustering of bacterial communities among treatment groups on dol 11 but not on the other days (Supplementary Figure S2). Specifically, the bacterial communities of both treated groups clustered apart from the bacterial communities of the control group (Figure 3). Total 16S rRNA gene copy numbers, in turn, were differently affected by the treatments, showing a depressing effect of the oral toltrazuril compared to the other two groups on dol 15 (*P* < 0.05; Supplementary Table S2).

**TABLE 1:** α-diversity indices by treatment groups and days of life.

**Day of life**	**Parenteral toltrazuril**	**Oral toltrazuril**	**Control**	**SEM**	**Treatment effect**
**A – Species richness**					
1	156	152	98	30.66	0.352
3	132	123	120	12.19	0.759
5	173	178	183	12.23	0.840
11	263^a^	237^a^	158^b^	18.72	**<0.001**
15	305	301	250	32.71	0.443
31	445	429	486	32.47	0.468
34	464	418	438	23.91	0.385
38	428	471	452	26.80	0.514
**B – Simpson index**					
1	0.744	0.702	0.814	0.0636	0.470
3	0.879	0.878	0.870	0.0188	0.933
5	0.920	0.930	0.929	0.0152	0.858
11	0.968^a^	0.966^a^	0.912^b^	0.0089	**<0.001**
15	0.965	0.943	0.960	0.0116	0.362
31	0.984	0.984	0.984	0.0015	0.916
34	0.985	0.982	0.986	0.0014	0.214
38	0.986	0.984	0.985	0.0015	0.642
**C – Shannon index**					
1	2.45	2.40	2.59	0.281	0.887
3	3.03	3.00	2.90	0.166	0.846
5	3.52	3.59	3.63	0.164	0.887
11	4.26^a^	4.24^a^	3.36^b^	0.133	**<0.001**
15	4.23	4.10	4.18	0.154	0.835
31	5.05	4.96	5.02	0.083	0.732
34	5.09	4.98	5.11	0.065	0.351
38	5.08	5.14	5.12	0.073	0.815

*Different superscript letters mark significant differences at *P* < 0.05. SEM: standard error of the mean. Bold numbers indicate statistically significant values (P ≤ 0.05).*

Alterations in relative taxa abundances confirmed results for α- and β-diversities, demonstrating that the greatest alterations in the fecal microbiota due to treatments were found on dol 11. Therefore, differences in the taxonomic composition are presented in detail here for the sampling days framing the acute phase of *C. suis* infection, dol 5 and 11 preweaning and dol 34, showing few treatment related effects post-weaning. The detailed results for the other sampling days pre- and post-weaning can be found in Supplementary Tables S3, S4. Alterations at the phylum level indicated that both the oral and parenteral administration of toltrazuril increased *Bacteroidetes* bacteria on dol 5 (Table 2), whereas this phylum was no longer affected by treatment on dol 11. Instead, the major phyla *Firmicutes* (+72.4%) and *Fusobacteria* (−75.7%) were affected by both toltrazuril treatments, whereas *Proteobacteria* (−73.4%) were only influenced by parenteral toltrazuril compared to the control (*P* < 0.05; Table 3). Also, less abundant phyla, i.e., *Actinobacteria, Synergistetes*, *Lentisphaera*, and *Euryarchaeota*, showed a treatment-related response on dol 11 (*P* < 0.05), whereby *Synergistetes* and *Lentisphaera* only increased with the orally administered toltrazuril compared to the other two groups.

**TABLE 2:** Differences in the relative abundances (>0.05% of all reads) of bacterial phyla and selected families and genera in feces of piglets on day 5 of life.

**Taxa**	**Parenteral toltrazuril**	**Oral toltrazuril**	**Control**	**SEM**	**Treatment, *P*-value**
**Phylum**					
p_*Firmicutes*	35.39	35.39	37.90	3.258	0.852
p_*Bacteroidetes*	38.50^a^	35.07^a^	28.60^b^	2.054	**0.011**
p_*Fusobacteria*	19.26	23.39	25.41	3.082	0.378
p_*Proteobacteria*	3.52	4.78	4.85	1.061	0.636
p_*Actinobacteria*	2.28	2.45	2.42	0.656	0.319
**Family**					
f_*Bifidobacteriaceae*	0.15^b^	0.70^a^	0.37^ab^	0.137	**0.016**
**Genus**					
g_*Bifidobacterium*	0.13^b^	0.63^a^	0.32^ab^	0.129	**0.019**
g_*Coprococcus*	0.37^a^	0.11^b^	0.13^b^	0.070	**0.019**
f_*Erysiphaceae*; g_	0.12^b^	0.18^ab^	0.33^a^	0.057	**0.050**

*Different superscript letters mark significant differences at *P* < 0.05. SEM: standard error of the mean. Bold numbers indicate statistically significant values (P ≤ 0.05).*

**TABLE 3:** Differences in the relative abundances (>0.05% of all reads) of bacterial phyla and selected families and genera in feces of piglets on day of life 11.

**Taxa**	**Parenteral toltrazuril**	**Oral toltrazuril**	**Control**	**SEM**	**Treatment, *P*-value**
**Phylum**					
p__*Firmicutes*	59.17^a^	59.06^a^	34.29^b^	4.714	**<0.001**
p__*Bacteroidetes*	27.73	25.08	26.64	3.730	0.886
p__*Fusobacteria*	7.96^b^	5.54^b^	27.83^a^	3.544	**<0.001**
p__Proteobacteria	2.62^b^	6.88^a^	9.84^a^	1.386	**0.003**
p__*Actinobacteria*	1.14	1.88	0.77	0.353	0.105
p__*Verrucomicrobia*	0.55	0.17	0.30	0.347	0.738
p__*Synergistetes*	0.089^b^	0.61^a^	0.039^b^	0.154	**0.029**
p__*Spirochaetes*	0.268	0.213	0.176	0.110	0.830
p__*Planctomycetes*	0.175	0.226	0.054	0.077	0.291
p__*Lentisphaerae*	0.138^b^	0.226^a^	0.049b	0.040	**0.016**
p__*Euryarchaeota*	0.100^a^	0.113^a^	0.007b	0.031	**0.044**
**Family**					
f_*Fusobacteriaceae*	7.73^b^	5.87^b^	26.86^a^	3.009	**<0.001**
f_*Ruminococcaceae*	16.79^a^	15.65^a^	4.15^b^	2.069	**<0.001**
f_*Lachnospiraceae*	11.30^a^	10.47^a^	4.19^b^	1.174	**<0.001**
f_*Veillonellaceae*	2.84^b^	3.18^b^	10.18^a^	1.792	**0.010**
f_*S24-7*	6.48^a^	4.08^a^	0.80^b^	0.894	**<0.001**
f_*Clostridiaceae*	3.97^a^	3.24^a^	0.94^b^	0.964	0.079
f_*Erysipelotrichaceae*	3.34^a^	3.58^a^	0.56^b^	0.584	**0.002**
f_*Enterobacteriaceae*	1.18^b^	4.72^a^	1.74^b^	0.928	**0.029**
f_*Campylobacteraceae*	0.44^ab^	0.003^b^	3.57^a^	1.118	0.065
f_*Bifidobacteriaceae*	0.099^b^	0.57^a^	0.10^b^	0.106	**0.006**
f_*Synergistaceae*	0.090^b^	0.64^a^	0^b^	0.144	**0.010**
f_*Victivallaceae*	0.13a^b^	0.22^a^	0.026^b^	0.036	**0.003**
f_*Methanobacteriaceae*	0.098^a^	0.11^a^	0.012^b^	0.029	**0.038**
f_*Micrococcaceae*	0.11^a^	0.058^ab^	0.010^b^	0.020	**0.006**
**Genus**					
g_*Fusobacterium*	7.75^b^	5.90^b^	26.88^a^	3.012	**<0.001**
g_*Oscillospira*	7.86^a^	6.91^a^	1.55^b^	1.099	**<0.001**
f_*Lachnospiraceae*; g_	5.76^a^	5.44^a^	1.27^b^	0.819	**<0.001**
g__*S24-7*	6.52^a^	4.10^a^	0.81^b^	0.899	**<0.001**
g_*Ruminococcus*	4.23^a^	4.32^a^	1.22^b^	0.905	**0.034**
f_*Ruminococcaceae*; g_	3.92^a^	3.62^a^	0.97^b^	0.845	**0.036**
g_*Escherichia*	1.16^b^	4.69^a^	1.73^b^	0.923	**0.029**
g_[*Eubacterium*]	2.24^a^	2.35^a^	0.27^b^	0.458	**0.005**
g_[*Ruminococcus*]	1.85^a^	1.89^a^	0.53^b^	0.326	**0.008**
g_*Anaerovibrio*	0.080^b^	0.056^b^	3.93^a^	1.077	**0.023**
g_*Rikenella*	1.30^a^	1.95^a^	0.51^b^	0.322	**0.015**
g_*SMB53*	1.69^a^	1.40^a^	0.23^b^	0.368	**0.020**
f_*Christensenellaceae*; g_	1.56^a^	1.30^a^	0.19^b^	0.403	**0.050**
g_*Blautia*	1.50^a^	0.59^b^	0.46^b^	0.291	**0.030**
g__*Butyricimonas*	1.12^a^	1.11^a^	0.36^b^	0.220	**0.030**
g__*Desulfovibrio*	0.69^a^	0.76^a^	0.17^b^	0.106	**<0.001**
o_*Bacteroidales*; f_; g_	0.84^a^	0.50^ab^	0.055^b^	0.213	**0.042**
f_*Mogibacteriaceae*; g_	0.46^b^	0.75^a^	0.088^a^	0.147	**0.014**
g_*Synergistes*	0.091^b^	0.64^a^	0^b^	0.145	**0.010**
g_*Bifidobacterium*	0.010^b^	0.44^a^	0.091^b^	0.079	**0.007**
g_*Dialister*	0.005^b^	0.056^b^	0.49^a^	0.067	**<0.001**
g_*Methanobrevibacter*	0.099^a^	0.11^a^	0.012^b^	0.029	**0.039**
g_*Rothia*	0.11^a^	0.059^ab^	0.011^b^	0.020	**0.006**

*Different superscript letters mark significant differences at *P* < 0.05. SEM: standard error of the mean. Bold numbers indicate statistically significant values (P ≤ 0.05).*

Although *Actinobacteria* were not affected by treatment at phylum level on dol 5 (Table 2), the family *Bifidobacteriaceae* and within it the genus *Bifidobacterium* were less abundant in the parenteral compared to the oral toltrazuril group (*P* < 0.05). In contrast, the genus *Coprococcus*, also belonging to *Actinobacteria*, was more abundant in the parenteral toltrazuril group compared to the two other groups. Treatment-related alterations in bacterial families and genera were more obvious on dol 11 (Table 3), showing increased abundances of *Fusobacteriaceae* and *Veillonellaceae* in feces of piglets from the control group. In contrast, other major bacterial families, such as *Ruminococcaceae*, *Lachnospiraceae*, *S24-7*, *Clostridiaceae* and *Erysipelotrichaceae* were more abundant in feces of piglets from both treatment groups compared to those in the control group (*P* < 0.05). The fecal abundances of *Enterobacteriaceae*, *Bifidobacteriaceae*, and *Synergistaceae*, in turn, were increased in feces of piglets receiving oral toltrazuril but not in the group with parenteral administration compared to the control on dol 11. Alterations in bacterial genera on dol 11 corresponded to changes observed at family level (Table 3). Treatment effects were absent on dol 15 and 31 but some treatment effects were detectable on dol 34 (Supplementary Tables S3, S4). Although no effects were detectable at phylum level on dol 34, *Clostridium*, one unclassified *Clostridiaceae* genus, SMB53 and *Turicibacter* were more highly abundant in the oral toltrazuril treatment group compared to the parenteral administration and the control groups (*P* < 0.05). In contrast, piglets in the control group had higher levels of *Roseburia* than the treatment groups, whereas the opposite response was observed for an unclassified RF13 genus.

**FIGURE 4:**
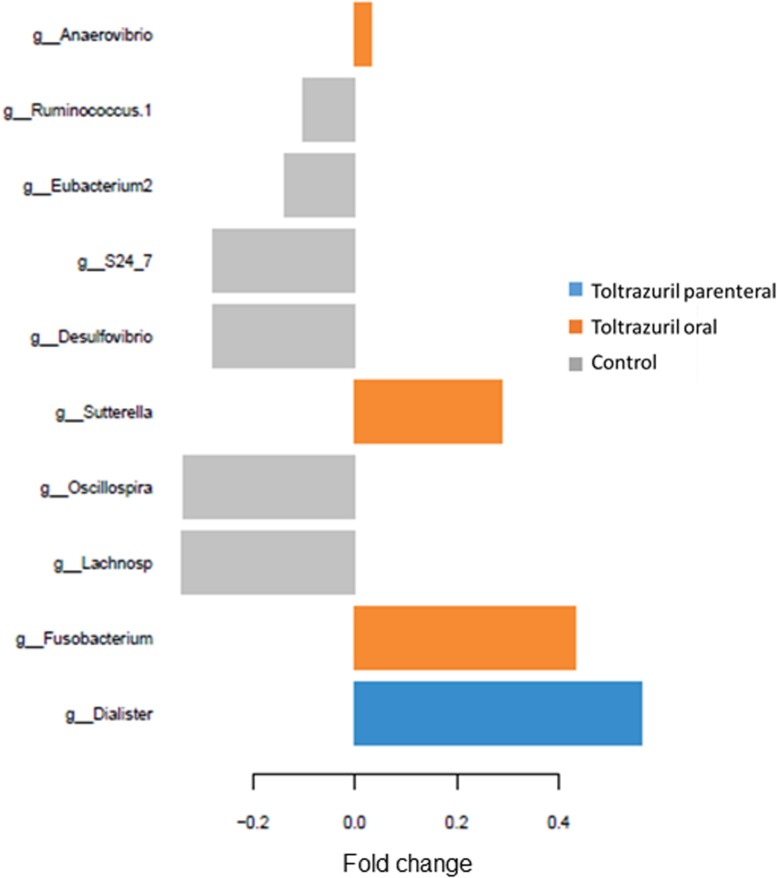
Loading plots of sparse partial least square-discriminant analysis (sPLS-DA) showing the most discriminant bacterial genera (relative genera abundance >0.05%). Positive fold changes indicate lower abundance; negative fold changes indicate higher abundance compared to the treatment means. Comp 1; component 1 of the sPLS-DA.

Moreover, sparse partial least square-discriminant analysis (sPLS-DA) was used to identify the most discriminant genera in feces in each treatment group on dol 11. An unclassified *Lachnospiraceae* and the genera *Oscillospira, Desulfobacterium, S24-7*, [*Eubacterium2*] and [*Ruminococcus1*] were most discriminant for the control group, whereas *Dialister* discriminated best with the parenteral toltrazuril treatment. By contrast, *Fusobacterium*, *Sutterella*, and *Anaerovibrio* were most influenced by the oral toltrazuril administration (Figure 4).

### Associations Among Clinical and Parasitological Parameters and Microbiota Composition

**FIGURE 5:**
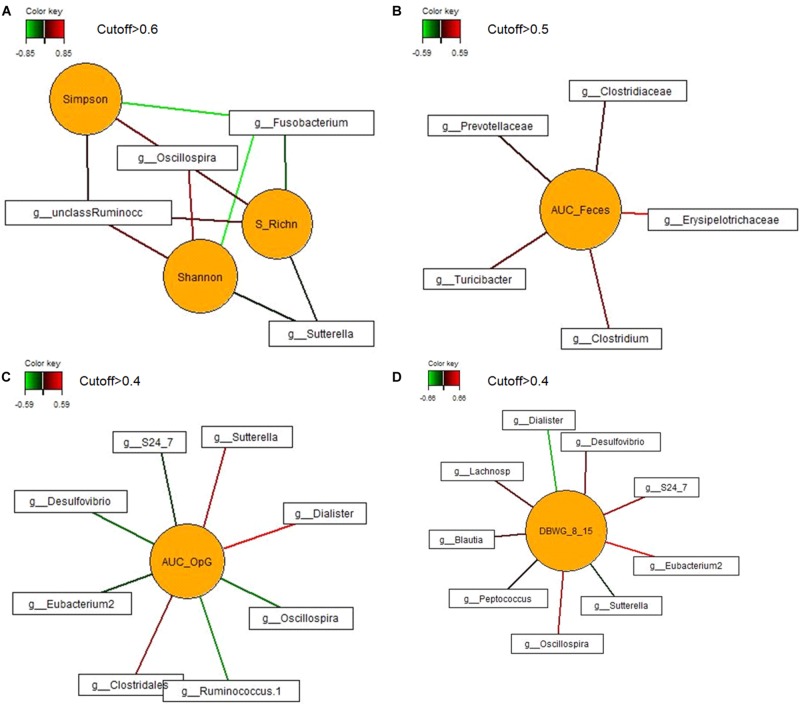
Associations between the most discriminant bacterial genera from dol 11 and **(A)** α-diversity (Shannon and Simpson) and species richness (S_RichN); **(B)** daily body weight gain between dol 8 and 15; **(C)** area under the curve for the fecal score from dols 5–18 (AUC_Feces); and **(D)** area under the curve of the oocysts per gram of feces from dol 5 to dol 18 (AUC_OpG). For statistical calculation of AUC see Shrestha et al. (2020). Covariations between the most relevant bacterial genera (relative abundance >0.05% of all reads), diversity and species richness, DBWG, AUC_Feces and AUC_OpG were established separately using sparse partial least squares regression and relevance networking. The networks are displayed graphically as nodes (parameters) and edges (biological relationship between nodes). The edge color intensity indicates the level of association: red = positive, green = negative. Only the strongest pairwise associations were projected. Unclass Ruminoc: unclassified *Ruminococcaceae* genus; Lachnosp: unclassified *Lachnospiraceae* genus.

In order to identify associations among bacterial genera and diversity indices, daily body weight gain and area under the curve for fecal score and for oocysts per gram feces on dol 11, first sparse partial least squares regression and relevance networking were performed. This analysis indicated negative relationships of *Sutterella* and *Fusobacterium* with species richness and diversity, whereas positive associations of *Oscillospira* and an unclassified genus of the family *Ruminococcaceae* with species richness and α-diversity indices were observed (Figure 5A). The area under the curve for the fecal score (as a parameter for diarrhea) was positively correlated with the genus *Clostridium* and the genus *Turicibacter* (Figure 5B). Negative relationships were indicated between the area under the curve for the oocysts per gram of feces (as a measure for the severity of infection) and five genera (*S24-7, Ruminococcus, Eubacterium, Oscillospira*, and *Desulfovibrio*), whereas positive relationships existed with an unclassified *Clostridiales* genus, *Sutterella*, and *Dialister* (Figure 5C). In addition five negative and four positive associations were determined for daily body weight gain of the piglets during the acute phase of infection (dol 8 to 15) including genera such as *S24-7*, *Blautia*, *Eubacterium*, *Oscillospira, Desulfovibrio, Sutterella*, and *Dialister* (Figure 5D).

## Discussion

Early life colonization of gut microbiota is vital to animal health, as it influences the microbial profile and intestinal health at later stages of life (Ke et al., 2019). Pathogenic protozoan parasites can interfere with the intestinal microbiota (Ras et al., 2015; Chabé et al., 2017; Huang et al., 2018a; Mammeri et al., 2019). In suckling piglets, *C. suis* commonly parasitizes the epithelial cells of the small intestine and is closely related to *Cystoisospora belli* of humans (Almeria et al., 2020) and is considered a major diarrheal pathogen (Joachim and Shrestha, 2020). Its synergism with toxigenic *Cp*A, another important cause of piglet diarrhea, has been previously demonstrated, as was the beneficial effect of early treatment with the anticoccidial drug toltrazuril in disrupting this synergism by effective parasite control (Mengel et al., 2012). However, little is known about the overall development of the intestinal microbiota of *C. suis* infected piglets and the influence of antiparasitic treatment.

The study design included a randomized (body weight-based) block design with inclusion of piglets from different litters into both groups to minimize litter effects on microbiota composition (Schokker et al., 2014). Due to the direct transmission mode of *C. suis* via oocysts excreted by infected litter mates, inclusion of an uninfected control group in the block design was not possible. Instead a group design with an untreated control group and two treatment groups was chosen to compare a group with the full development of the parasite (and subsequent excretion of oocysts) with groups where the development of the parasite and the clinical disease induced by it were prevented, assuming that the influence of the parasite on the intestinal microbial composition would be minimized.

In this study, 16S rRNA gene sequencing was used to determine the effects of parenteral and oral toltrazuril treatments on the development of the fecal microbiota in *C. suis* infected piglets during the suckling and early post-weaning period. Alterations in the acute phase of infection were mostly present on dol 11, whereby both toltrazuril treatments prevented the loss of bacterial species and diversity compared to piglets in the control group, which showed typical clinical signs of *C. suis* infection. Moreover, it was obvious that both toltrazuril treatments, parenteral and oral, affected the fecal taxa composition largely identically. This led to the assumption that alterations on the fecal microbiota after toltrazuril treatments might be due to an indirect effect via interruption of parasite development and subsequent maintenance of small intestinal functions during the acute phase of infection.

The present results confirmed previously reported age-related patterns in the fecal microbiota composition (Chen et al., 2017; Guevarra et al., 2019; Wang et al., 2019) beginning with more aero-tolerant taxa (e.g., *Escherichia* followed by *Bacteroides* and *Fusobacterium*) in the first days of life toward a complex and diverse community post-weaning, thereby contributing to the increase in species richness and evenness with age. As the intestinal colonization mainly takes place post-farrowing, it was expected that the composition on the first dol largely differed from that of the other sampling days. The bacterial community from dol 3 clustered apart from those of dols 5, 11, and 15; however, the shifts in the bacterial community from pre- to post-weaning were more drastic, emphasizing the importance of the type of ingested food. Piglets had access to creep feed from the second week of life but it was expected that the intake of solid feed was very low at the beginning and differed among piglets and litters. This was reflected by the greater inter-individual differences before weaning which converged post-weaning showing a greater uniformity among the microbial communities of different piglets. Similar patterns in α- and β-diversities in the gut microbiota of piglets have been reported previously (Chen et al., 2017). Therefore, the present results confirmed the progressing microbial maturation during the lactation period, which, after the complete transition to solid feed, led to the gradual establishment of a more stable community post-weaning (Guevarra et al., 2019; Wang et al., 2019). A sharp decrease in the relative abundances of *Fusobacterium* and *Bacteroides* was found from the suckling to post-weaning period. *Bacteroides* species utilize milk oligosaccharides and host-derived glycans as carbon sources and often decline post-weaning (Frese et al., 2015; Marcobal et al., 2011). Consistent with this, members of *Clostridiaceae*, *Lactobacillus*, and *Prevotella* increased in their abundance after weaning, reflecting the dietary change from sow’s milk to a solid cereal-based diet, bacterial substrate preferences and metabolic capabilities (Schwab and Gänzle, 2011; Flint et al., 2012; Motta et al., 2019; Wang et al., 2019).

The gut microbiota plays an essential role in the resistance to colonization by enteric pathogens, including parasitic protozoa, in the gut (Kamada et al., 2013; Hirt, 2019). Therefore, disruption in the compositional development of gut microbiota in early life might also alter the pathophysiology of parasitic infection either by promoting infection or by conferring resistance (Partida-Rodríguez et al., 2017; Huang et al., 2018b; Oliveira and Widmer, 2018). The decrease in diversity and large alterations in taxonomic composition on dol 11 may support previous findings that protozoan infections can disrupt the enteric microbiota. As a consequence, the resulting dysbiosis may alter the clinical and pathological outcome of parasitic infections, as previously shown for coccidiosis in chickens (Macdonald et al., 2017; Huang et al., 2018a) and mice (Huang et al., 2018b) and cryptosporidiosis in mice and humans (Chappell et al., 2016; Mammeri et al., 2019).

Disruption of the intestinal epithelium caused by *C. suis* infection can induce diarrhea in the absence of other enteropathogens (Harleman and Meyer, 1985; Vítovec and Koudela, 1990) but is aggravated by co-infection with other enteric pathogens which are also common in conventional pigs (Mengel et al., 2012). Compared to gnotobiotic piglets, *C. suis* infection markedly influenced early mortality in piglets that received bacterial flora derived from intestinal contents of conventional piglets with clinical coccidiosis, indicating that gut microbiota have opportunistic rather than synergistic function in *C. suis* infected piglets (Harleman and Meyer, 1985). Since the majority of the life cycle of *C. suis* takes place in intestinal epithelial cells and direct interactions of extracellular parasite stages with the gut microbiota are short and transient, perturbation of the microbial population is likely due to the damage to the intestinal epithelium caused by replicating parasites (Mundt et al., 2006, 2007), rather than by direct interaction of the parasite and the bacterial population. For this reason, present relevance networks for OpG excretion and fecal score may support the previously observed correlation between clinical coccidiosis, infection with *Cp*A and early toltrazuril treatment in suckling piglets (Mengel et al., 2012) and chickens (Alnassan et al., 2013), thereby confirming the assumption that coccidia might create a favorable environment for colonization by opportunistic gut pathogens.

The pig gut microbiota represents a highly complex and dynamic microbial community which is influenced by many factors including the environment, age, diet and breed (Crespo-Piazuelo et al., 2019). Since all animals in the present study were derived from three litters that were fed the same diet and were housed under identical conditions, plus that a random block design was applied to assign piglets to the different treatment groups to account for litter effects, the observed differences in bacterial diversity and taxonomy can be assumed to be the consequences of toltrazuril applications. Following infection and treatment, the greatest treatment-related differences in the gut microbiota occurred on dol 11, when all animals from the control group had shed oocysts at least for 1 day. Interestingly, before and after that, piglets from the different groups had largely similar fecal microbial patterns. Notably, both toltrazuril treatments prevented the loss of bacterial diversity as was observed in the piglets of the control group on dol 11. This is in accordance with previous studies in which lowest α-diversities were reported in *Eimeria* challenged chickens (Stanley et al., 2014; Wu et al., 2014). In general, bacterial species richness and diversity often reflect stability and resilience of the gut ecosystem and are therefore considered as potential markers for gut health (Vandeputte et al., 2016). Several reasons for our observations are conceivable. First, the small effect of toltrazuril treatments on the other dols hints at a high plasticity of the fecal microbiota in these young animals. Second, given that dol 11 corresponded to the day of the maximum prevalence of diarrhea and a decline of oocyst excretion in the control group, *C. suis* infection-related loss of gut integrity and function as well as increased intestinal disruption might have caused the loss of diversity in the control piglets. Conversely, both toltrazuril treatments probably maintained the gut integrity by inhibiting the establishment of *C. suis* and subsequent dysbiosis associated with the proliferation of specific bacteria and thus maintained a high bacterial diversity in the gastrointestinal tract. This assumption is confirmed by the finding that the toltrazuril treatment effects on taxonomic composition disappeared on dol 15, supporting an indirect effect of the toltrazuril on the gut microbiota via maintenance of the host physiology. Third, microbial transitions following inclusion of pre-starter diet at dol 14 (pre-weaning) and weaning stress might have masked treatment effects at later time points. Notably, although treatment effects were absent for the next two sampling days pre- and post-weaning (dol 15 and 31), it can be speculated whether the oral toltrazuril administration caused certain long-lasting effects on the host physiology (e.g., mucin production), as mucin-degrading taxa, such as *Clostridium* and *Turicibacter*, were increased with this treatment post-weaning. Since the present study was based on fecal data, however, we can only speculate about the cause-and-effect relationships that may have occurred intestinally.

Toltrazuril is a coccidiocidal drug that is almost completely absorbed from the intestine following oral administration (Mehlhorn and Greif, 2016), and parenteral (intramuscular) administration of toltrazuril resulted in more sustained concentrations in jejunal and ileal tissues and contents compared to oral application (Karembe et al., 2019). Although drug concentrations at the predilection site of *C. suis* is crucial for its pharmacological effects, higher drug concentration for longer period might also have an indirect effect on other local gut microbiota. Since *Escherichia* spp. rely on other obligate gut anaerobes for mono- and disaccharides needed for their growth (Conway and Cohen, 2015), inhibition of these anaerobes might have resulted lower abundances of *Escherichia* in piglets that received parenteral toltrazuril compared to those who received oral toltrazuril on dol 11.

Whether the promotion of the phylum *Bacteroidetes* as a whole in both treatment groups on dol 5 was related to a direct effect of toltrazuril on the gut microbiota or an indirect effect via maintaining epithelial integrity cannot be differentiated by the present results. However, the reduced abundance of the genus *Bifidobacterium* and the higher abundance of the genus *Coprococcus* in the parenteral toltrazuril group compared to the oral toltrazuril group on dol 5 indicated other, possibly more direct effects of toltrazuril or its metabolites on the gut microbiota, which needs further investigation.

Treatment-associated bacterial shifts were largely evident on dol 11 with a marked increase in the abundance of *Fusobacteriaceae* and *Veillonellaceae* in the control group. Interestingly, increased abundance of the genus *Fusobacterium* coincided with the highest prevalence of diarrhea in control piglets on dol 11. Relevance networking also revealed that, besides *Sutterella*, *Fusobacterium* was negatively associated with α-diversity as shown previously (Vandeputte et al., 2016; Kwon et al., 2019). *Fusobacterium* spp. are a gram-negative bacilli, and increased relative abundance has been reported in piglets with diarrhea (Hermann-Bank et al., 2015; Yang et al., 2017) and calves with cryptosporidiosis (Ichikawa-Seki et al., 2019). The altered composition of glycoconjugates in villous enterocytes, enhanced mucus secretion by goblet cells and damage to microvilli during acute *C. suis* infection (Kudweis et al., 1989; Kudweis et al., 1990; Choi et al., 2003) may promote intestinal colonization by *Fusobacterium* and foster the development of severe diarrhea. Lower abundance of *Fusobacterium* (as demonstrated by sPLS-DA) and absence of diarrhea in toltrazuril treated piglets compared to the control piglets advocate a significant contribution of this genus to the occurrence of diarrhea in the presence of *C. suis*. Similarly, significantly higher abundance of *Veillonellaceae* in the control group might be associated with an intense inflammatory response following destruction of the epithelial lining by *C. suis*. An increased abundance of *Veillonellaceae* was positively correlated with the incidence of inflammatory bowel disease in humans (Gevers et al., 2017), although the exact underlying mechanism is yet to be elucidated.

Loss of intestinal integrity, increased motility and changes in nutrient flows may be the reasons for the greatly reduced abundances of some of the major anaerobic commensal bacteria such as *Ruminococcaceae, Lachnospiraceae, Bacteroidales S24-7, Clostridiaceae*, and *Erysipelotrichaceae* in the control compared to the treated groups. For instance, members of *Ruminococcaceae* and *Lachnospiraceae* are main contributors to butyrate biosynthesis (Flint et al., 2012), and their increased abundance has been associated with increased cell proliferation and recovery of intestinal morphology (Zhong et al., 2019). Detailed studies on how toltrazuril assist in preventing *C. suis* induced gut dysbiosis will help to develop strategies for modulation of gut microbiota to restore homeostasis and promote piglet health in affected litters.

Sparse partial least square-discriminant analysis identified specific genera that could potentially contribute to differentiate the treatment groups. Apart from *Fusobacterium*, both treated groups displayed a lower relative abundance of the most discriminating genera *Dialister, Sutterella*, and *Anaerovibrio*. *Dialister* and *Sutterella* have been associated with intestinal inflammatory disorders (Tito et al., 2017). Therefore, lower relative abundances of these genera in treated groups also advocate the role of toltrazuril in maintaining gut homeostasis in infected piglets, probably indirectly by inhibition of intracellular parasite replication and damage of epithelium. In line with that, relevance networking suggested that the abundances of *Sutterella* and *Dialister* were negatively associated with daily body weight gain during the acute phase of infection, potentially supporting a role of these taxa during acute gut inflammation. In contrast, *Eubacterium* and *Oscillospira* were positively correlated with body weight gain during the acute phase of infection. *Oscillospira* is often reported to decline in inflammatory diseases (Gophna et al., 2017) and both taxa have capacities to utilize resistant starch and are important butyrate producing genera (Schwab and Gänzle, 2011; Gophna et al., 2017), which may promote intestinal integrity and indirectly support the physical development of the piglet itself. Further studies are required to understand how such microbial modulations contribute to body weight development in the suckling piglet.

The area under the curve for the fecal score (indicating diarrhea) was positively related to *Clostridium and Turicibacter* on dol 11, both of which are commensals in the porcine gut. Nevertheless, *Clostridium* has been associated with gastrointestinal disorders and diarrhea in several mammalian species, including humans (Larson and Borriello, 1988; Uzal and McClane, 2011; Chan et al., 2012) and suckling piglets (Driesen et al., 1993), showing higher relative abundance in diarrheic compared to healthy individuals. Positive associations of the genera *Sutterella, Dialister* and *Clostridiales* with the area under the curve for oocyst per gram feces in the present study (indicating the severity of infection) also indicated that the gut microbiota in general is probably considerably affected by *C. suis* infection. By contrast, repeated supplementation of probiotic bacterial cocktail immediately after birth had no influence on fecal consistency and amount and duration of oocyst excretion in *C. suis* infected piglets (Unterweger et al., 2018); therefore, this influence appears largely one-sided. However, further studies are necessary to elucidate the underlying role of specific microbial populations in the establishment and overall consequences of cystoisosporosis.

## Conclusion

With increasing pressure to reduce the use of antimicrobials in production animals, there is a growing interest to better understand the shifts in microbial composition and structure during individual development, also in the presence of enteropathogens. The present study showed that irrespective of the application form, treatment with toltrazuril suppressed the development of cystoisosporosis and therefore supported maintenance of gut integrity and functioning that may have prevented the loss of diversity and alterations in the microbial community during the acute phase of infection as was observed in the control group suffering from cystoisosporosis. The present results also showed that after the acute phase of infection and directly post-weaning, only minor differences in the microbiota composition among treatment groups existed, supporting a high plasticity of the fecal microbiota with restoration of the intestinal homeostasis after the acute phase of infection.

## Data Availability Statement

Raw sequence reads were uploaded to the NCBI BioProject databank (PRJNA599311). Further data on the *C. suis* challenge and the influence of treatment not mentioned here are published in: Joachim A, Guerra N, Hinney B, Hodžić A, Karembe H, Shrestha A, Sperling D. Efficacy of injectable toltrazuril-iron combination product and oral toltrazuril against early experimental infection of suckling piglets with *Cystoisospora suis*. Parasites and Vectors. 2019 May 28;12(1):272. 10.1186/s13071-019-3527-3.

## Ethics Statement

This study was designed under GSP conditions in compliance with Directive 2001/82/EC amended by the Directive 2004/28/EC and by the Directive 2009/09/EC of the European Parliament and of the Council. All procedures involving piglets were approved by the Animal Ethics Committee of the University of Veterinary Medicine Vienna and the national authority according to §26ff of Animal Experiments Act, Tierversuchsgesetz 2012-TVG 2012 (license number: BMWF-68.205/0034-WF/V/3b/2016; Austrian Federal Ministry of Science, Health and Economy).

## Author Contributions

BM-Z, DS, HK, and AJ designed the study. AS carried out the animal trial and sampling, AJ and AS analyzed the clinical and parasitological parameters. BM-Z and SK carried out the microbiota analyses. BM-Z performed the bioinformatics and statistics. AS, BM-Z, and AJ drafted the manuscript. All authors read and approved of the final version of the manuscript.

## Conflict of Interest

DS and HK are employees of Ceva Santé Animale, France, the study sponsor. The remaining authors the research was conducted in the absence of any commercial or financial relationships that could be construed as a potential conflict of interest.

## Footnotes

1 https://cutadapt.readthedocs.org/

## Supplementary Material

FIGURE S1 Mean fecal scores (all groups) and oocyst excretion (ln of oocysts per gram [OPG]; control only) during the study period. All groups: infection with *C. suis* on the first day of life. Groups: Parenteral Toltra (treatment with parenteral toltrazuril on the second day of life), Oral Toltra (treatment with oral toltrazuril on the fourth day of life), Control: no treatment.

FIGURE S2 Non-metric multidimensional scaling plot of pairwise Bray-Curtis dissimilarities between bacterial communities at genus level in feces of suckling and weaned piglets (>0.01% relative abundance) showing the treatment effects on all sampling time points (day of life: 1, 3, 5, 11, 15, 31, 34, and 38; stress: 0.1067) between different groups (group A = parenteral toltrazuril, group B = oral toltrazuril, group C = untreated control group).

TABLE S1 Comparison of groups: Oocyst excretion, diarrhea, body weight gain and concomitant infections. OpG: oocysts per gram of feces, dol: day of life, g: gram.

TABLE S2 Total bacterial 16S rRNA gene copy numbers by treatments groups and days of life. Different superscript letters mark significant differences at *P* < 0.05. SEM: standard error of the mean.

TABLE S3 Differences in the relative abundances (>0.01% of all reads) of bacterial phyla in feces of piglets on days 1, 3, 5, 11, 15, 31, 34, and 38 of life. Different superscript letters mark significant differences at *P* < 0.05. SEM: standard error of the mean.

TABLE S4 Differences in the relative abundances (>0.01% of all reads) of bacterial genera in feces of piglets on days 1, 3, 5, 11, 15, 31, 34, and 38 of life. Different superscript letters mark significant differences at *P* < 0.05. SEM: standard error of the mean.

## References

[B1] Almeria S. Cinar H. N. Dubey J. P. (2020). “Coccidiosis in humans,” in *Coccidiosis in Livestock, Poultry, Companion Animals, and Humans*, ed. Dubey J. P. (Boca Raton: CRC Press), 267–312.

[B2] Alnassan A. A. Shehata A. A. Kotsch M. Schrödl W. Krüger M. Daugschies A. (2013). Efficacy of early treatment with toltrazuril in prevention of coccidiosis and necrotic enteritis in chickens. *Avian Pathol.* 42 482–490. 10.1080/03079457.2013.823476 23941631

[B3] Barta J. R. Schrenzel M. D. Carreno R. Rideout B. A. (2005). The genus *Atoxoplasma* (Garnham 1950) as a junior objective synonym of the genus *Isospora* (Schneider 1881) species infecting birds and resurrection of *Cystoisospora* (Frenkel 1977) as the correct genus for *Isospora* species infecting mammals. *J. Parasitol.* 91 726–727. 10.1645/GE-3341.1 16108579

[B4] Callahan B. J. McMurdie P. J. Rosen M. J. Han A. W. Johnson A. J. A. Holmes S. P. (2016). DADA2: high-resolution sample inference from Illumina amplicon data. *Nat. Methods* 13 581–583. 27214047 10.1038/nmeth.3869 PMC4927377

[B5] Chabé M. Lokmer A. Ségurel L. (2017). Gut protozoa: friends or foes of the human gut microbiota? *Trends Parasitol.* 33 925–934. 10.1016/j.pt.2017.08.005 28870496

[B6] Chan G. Farzan A. Soltes G. Nicholson V. M. Pei Y. Friendship R. (2012). The epidemiology of *Clostridium perfringens* type A on Ontario swine farms, with special reference to cpb2-positive isolates. *BMC Vet. Res.* 8:156 10.1186/1746-6148-8-156 PMC3503845 22947389

[B7] Chappell C. L. Darkoh C. Shimmin L. Farhana N. Kim D. K. Okhuysen P. C. (2016). Fecal indole as a biomarker of susceptibility to *Cryptosporidium* infection. *Infect. Immun.* 84 2299–2306. 10.1128/iai.00336-16 27245413 PMC4962629

[B8] Chen L. Xu Y. Chen X. Fang C. Zhao L. Chen F. (2017). The maturing development of gut microbiota in commercial piglets during the weaning transition. *Front. Microbiol.* 8:1688 10.3389/fmicb.2017.01688 PMC5591375 28928724

[B9] Choi B. Y. Sohn Y. S. Choi C. Chae C. (2003). Lectin histochemistry for glycoconjugates in the small intestines of piglets naturally infected with *Isospora suis*. *J. Vet. Med. Sci.* 65 389–392. 10.1292/jvms.65.389 12679572

[B10] Conway T. Cohen P. S. (2015). Commensal and pathogenic *Escherichia coli* metabolism in the gut. *Microbiol. Spectr.* 3:10.1128/microbiolsec.MB–0006–2014. 10.1128/microbiolspec.MBP-0006-2014 PMC4510460 26185077

[B11] Crespo-Piazuelo D. Migura-Garcia L. Estellé J. Criado-Mesas L. Revilla M. Castelló A. (2019). Association between the pig genome and its gut microbiota composition. *Sci. Rep.* 9:8791 10.1038/s41598-019-45066-6 PMC6584621 31217427

[B12] Dou S. Gadonna-Widehem P. Rome V. Hamoudi D. Rhazi L. Lakhal L. (2017). Characterisation of early-life fecal microbiota in susceptible and healthy pigs to post-weaning diarrhoea. *PLoS One* 12:e0169851 10.1371/journal.pone.0169851 PMC5225014 28072880

[B13] Driesen S. J. Carland P. G. Fahy V. A. (1993). Studies on preweaning piglet diarrhoea. *Aust. Vet. J.* 70 259–262. 8368968 10.1111/j.1751-0813.1993.tb08044.x

[B14] Flint H. J. Scott K. P. Duncan S. H. Louis P. Forano E. (2012). Microbial degradation of complex carbohydrates in the gut. *Gut Microbes* 3 289–306. 10.4161/gmic.19897 22572875 PMC3463488

[B15] Frese S. A. Parker K. Calvert C. C. Mills D. A. (2015). Diet shapes the gut microbiome of pigs during nursing and weaning. *Microbiome* 3:28 10.1186/s40168-015-0091-8 PMC4499176 26167280

[B16] Gevers D. Kugathasan S. Knights D. Kostic A. D. Knight R. Xavier R. J. (2017). A microbiome foundation for the study of Crohn‘s disease. *Cell Host Microbe* 21 301–304. 10.1016/j.chom.2017.02.012 28279336 PMC5684696

[B17] Gophna U. Konikoff T. Nielsen H. B. (2017). *Oscillospira* and related bacteria – from metagenomic species to metabolic features. *Environ. Microbiol.* 19 835–841. 10.1111/1462-2920.13658 28028921

[B18] Guevarra R. B. Lee J. H. Lee S. H. Seok M.-J. Kim D. W. Kang B. N. (2019). Piglet gut microbial shifts early in life: causes and effects. *J. Anim. Sci. Biotechnol.* 10:1 10.1186/s40104-018-0308-3 PMC6330741 30651985

[B19] Harder A. Haberkorn A. (1989). Possible mode of action of toltrazuril: studies on two *Eimeria* species and mammalian and *Ascaris suum* enzymes. *Parasitol. Res.* 76 8–12. 2560189 10.1007/BF00931064

[B20] Harleman J. H. Meyer R. C. (1984). Life cycle of *Isospora suis* in gnotobiotic and conventionalized piglets. *Vet. Parasitol.* 17 27–39. 6543059 10.1016/0304-4017(84)90062-1

[B21] Harleman J. H. Meyer R. C. (1985). Pathogenicity of *Isospsora suis* in gnotobiotic and conventionalised piglets. *Vet. Rec.* 116 561–565. 10.1136/vr.116.21.561 4024419

[B22] Hermann-Bank M. L. Skovgaard K. Stockmarr A. Strube M. L. Larsen N. Kongsted H. (2015). Characterization of the bacterial gut microbiota of piglets suffering from new neonatal porcine diarrhoea. *BMC Vet. Res.* 11:139 10.1186/s12917-015-0419-4 PMC4476181 26099928

[B23] Hirt R. P. (2019). Mucosal microbial parasites/symbionts in health and disease: an integrative overview. *Parasitology* 146 1109–1115. 10.1017/S0031182019000647 31378213 PMC6817359

[B24] Huang G. Tang X. Bi F. Hao Z. Han Z. Suo J. (2018a). *Eimeria tenella* infection perturbs the chicken gut microbiota from the onset of oocyst shedding. *Vet. Parasitol.* 258 30–37. 10.1016/j.vetpar.2018.06.005 30105975

[B25] Huang G. Zhang S. Zhou C. Tang X. Li C. Wang C. (2018b). Influence of *Eimeria falciformis* infection on gut microbiota and metabolic pathways in mice. *Infect. Immun.* 86:e00073-18 10.1128/IAI.00073-18 PMC5913850 29440368

[B26] Ichikawa-Seki M. Motooka D. Kinami A. Murakoshi F. Takahashi Y. Aita J. (2019). Specific increase of *Fusobacterium* in the faecal microbiota of neonatal calves infected with *Cryptosporidium parvum*. *Sci. Rep.* 9:12517 10.1038/s41598-019-48969-6 PMC6715637 31467354

[B27] Joachim A. Altreuther G. Bangoura B. Charles S. Daugschies A. Hinney B. (2018a). W A A V P guideline for evaluating the efficacy of anticoccidials in mammals (pigs, dogs, cattle, sheep). *Vet. Parasitol.* 253 102–119. 10.1016/j.vetpar.2018.02.029 29604993

[B28] Joachim A. Guerra N. Hinney B. Hodžić A. Karembe H. Shrestha A. (2019). Efficacy of injectable toltrazuril-iron combination product and oral toltrazuril against early experimental infection of suckling piglets with *Cystoisospora suis*. *Parasit. Vectors* 12:272 10.1186/s13071-019-3527-3 PMC6540551 31138327

[B29] Joachim A. Mundt H. C. (2011). Efficacy of sulfonamides and Baycox^®^ against *Isospora suis* in experimental infections of suckling piglets. *Parasitol. Res.* 109 1653–1659. 10.1007/s00436-011-2438-9 21556685

[B30] Joachim A. Ruttkowski B. Sperling D. (2018b). Detection of *Cystoisospora* suis in faeces of suckling piglets – when and how? A comparison of methods. *Porcine Health Manag.* 4:20 10.1186/s40813-018-0097-2 PMC6145109 30250747

[B31] Joachim A. Shrestha A. (2020). “Coccidiosis of pigs,” in *Coccidiosis in Livestock, Poultry, Companion Animals, and Humans*, ed. Dubey J. P. (Boca Raton, FL: CRC Press), 125–145.

[B32] Joachim A. Shrestha A. Freudenschuss B. Palmieri N. Hinney B. Karembe H. (2018c). Comparison of an injectable toltrazuril-gleptoferron (Forceris^®^) and an oral toltrazuril (Baycox^®^) + injectable iron dextran for the control of experimentally induced piglet cystoisosporosis. *Parasit. Vectors* 11:206 10.1186/s13071-018-2797-5 PMC5870915 29580269

[B33] Kamada N. Chen G. Y. Inohara N. Núñez G. (2013). Control of pathogens and pathobionts by the gut microbiota. *Nat. Immunol.* 14 685–690. 10.1038/ni.2608 23778796 PMC4083503

[B34] Karembe H. Magnier R. Peyrrou M. P. Guerra N. Vasek J. Smola J. (2019). “Plasma disposition kinetics and distribution of toltrazuril and its main metabolite in intestinal tissues and contents of piglets after oral and intramuscular administrations,” in *Proceedings of the 11 th European Symposium of Porcine Health Management 22-24 May 2019*, Utretch.

[B35] Ke S. Fang S. He M. Huang X. Yang H. Yang B. (2019). Age-based dynamic changes of phylogenetic composition and interaction networks of health pig gut microbiome feeding in a uniformed condition. *BMC Vet. Res.* 15:172 10.1186/s12917-019-1918-5 PMC6534858 31126262

[B36] Klinsoda J. Vötterl J. Zebeli Q. Metzler-Zebeli B. U. (2019). Lactic acid treatment of cereals and dietary phytase modified fecal microbiome composition without affecting expression of virulence factor genes in growing pigs. *Front. Microbiol.* 10:2345 10.3389/fmicb.2019.02345 PMC6808178 31681210

[B37] Koudela B. Kučerová ŠP. (1999). Role of acquired immunity and natural age resistance on course of *Isospora suis* coccidiosis in nursing piglets. *Vet. Parasitol.* 82 93–99. 10.1016/S0304-4017(99)00009-6 10321581

[B38] Kudweis M. Lojda Z. Koudela B. Vítovec J. (1989). Mucus synthesis in the goblet cells of the small intestine in experimental infection with the coccidium *Isospora suis* in piglets. *Vet. Med. (Praha)* 34 727–734. 2631376

[B39] Kudweis M. Lojda Z. Vítovec J. Koudela B. (1990). Activity of mucosubstances and the goblet cell count in the large intestine of piglets infected with the coccidium, *Isospora suis*. *Vet. Med. (Praha)* 35 21–29. 2333678

[B40] Kwon H. J. Lim J. H. Kang D. Lim S. Park S. J. Kim J. H. (2019). Is stool frequency associated with the richness and community composition of gut microbiota? *Intest. Res.* 17 419–426. 10.5217/ir.2018.00149 30704159 PMC6667361

[B41] Larson H. E. Borriello S. P. (1988). Infectious diarrhea due to *Clostridium perfringens*. *J. Infect. Dis.* 157 390–391. 10.1093/infdis/157.2.390 2891779

[B42] Lindsay D. S. Current W. L. Taylor J. R. (1985). Effects of experimentally induced *Isospora suis* infection on morbidity, mortality and weight gains in nursing pigs. *Am. J. Vet. Res.* 46 1511–1512. 4026033

[B43] Lindsay D. S. Dubey J. P. Blagburn B. L. (1997). Biology of *Isospora* spp. from humans, nonhuman primates, and domestic animals. *Clin. Microbiol. Rev.* 10 19–34. 8993857 10.1128/cmr.10.1.19 PMC172913

[B44] Macdonald S. E. Nolan M. J. Harman K. Boulton K. Hume D. A. Tomley F. M. (2017). Effects of *Eimeria tenella* infection on chicken caecal microbiome diversity, exploring variation associated with severity of pathology. *PLoS One* 12:e0184890 10.1371/journal.pone.0184890 PMC5608234 28934262

[B45] Mammeri M. Chevillot A. Thomas M. Julien C. Auclair E. Pollet T. (2019). *Cryptosporidium parvum*-infected neonatal mice show gut microbiota remodelling using high-throughput sequencing analysis: preliminary results. *Acta Parasitol.* 64 268–275. 10.2478/s11686-019-00044-w 30915719

[B46] Marcobal A. Barboza M. Sonnenburg Erica D. Pudlo N. Martens Eric C. Desai P. (2011). *Bacteroides* in the infant gut consume milk oligosaccharides via mucus-utilization pathways. *Cell Host Microbe* 10 507–514. 10.1016/j.chom.2011.10.007 22036470 PMC3227561

[B47] Mehlhorn H. Greif G. (2016). “Baycox,” in *Encyclopedia of Parasitology: A-M*, ed. Mehlhorn H. (Berlin: Springer), 299–300.

[B48] Mengel H. Krüger M. Krüger M. U. Westphal B. Swidsinski A. Schwarz S. (2012). Necrotic enteritis due to simultaneous infection with *Isospora suis* and clostridia in newborn piglets and its prevention by early treatment with toltrazuril. *Parasitol. Res.* 110 1347–1355. 10.1007/s00436-011-2633-8 21968954

[B49] Metzler-Zebeli B. U. Newman M. A. Grüll D. Zebeli Q. (2019). Functional adaptations in the cecal and colonic metagenomes associated with the consumption of transglycosylated starch in a pig model. *BMC Microbiol.* 19:87 10.1186/s12866-019-1462-2 PMC6498482 31046662

[B50] Meyer C. Joachim A. Daugschies A. (1999). Occurrence of *Isospora suis* in larger piglet production units and on specialized piglet rearing farms. *Vet. Parasitol.* 82 277–284. 10.1016/S0304-4017(99)00027-8 10384903

[B51] Motta V. Luise D. Bosi P. Trevisi P. (2019). Faecal microbiota shift during weaning transition in piglets and evaluation of AO blood types as shaping factor for the bacterial community profile. *PLoS One* 14:e0217001 10.1371/journal.pone.0217001 PMC6522051 31095619

[B52] Mundt H. C. Daugschies A. Wüstenberg S. Zimmermann M. (2003). Studies on the efficacy of toltrazuril, diclazuril and sulphadimidine against artificial infections with *Isospora suis* in piglets. *Parasitol. Res.* 90 S160–S162. 10.1007/s00436-003-0927-1 12928891

[B53] Mundt H. C. Joachim A. Becka M. Daugschies A. (2006). *Isospora suis*: an experimental model for mammalian intestinal coccidiosis. *Parasitol. Res.* 98 167–175. 10.1007/s00436-005-0030-x 16323027

[B54] Mundt H. C. Mundt-Wüstenberg S. Daugschies A. Joachim A. (2007). Efficacy of various anticoccidials against experimental porcine neonatal isosporosis. *Parasitol. Res.* 100 401–411. 10.1007/s00436-006-0314-9 17048000

[B55] Muyzer G. de Waal E. C. Uitterlinden A. G. (1993). Profiling of complex microbial populations by denaturing gradient gel electrophoresis analysis of polymerase chain reaction-amplified genes coding for 16S rRNA. *Appl. Environ. Microbiol.* 59 695–700. 7683183 10.1128/aem.59.3.695-700.1993 PMC202176

[B56] Oksanen J. Blanchet F. G. Friendly M. Kindt R. Legendre P. McGlinn D. (2018). *Vegan: Community Ecology Package. R Package Version 2.5-2.* Available online at: https://cran.r-project.org (accessed April 24, 2020).

[B57] Oliveira B. C. M. Widmer G. (2018). Probiotic product enhances susceptibility of mice to Cryptosporidiosis. *Appl. Environ. Microbiol.* 84 e1408–e1418. 10.1128/aem.01408-18 PMC6193388 30171003

[B58] Partida-Rodríguez O. Serrano-Vázquez A. Nieves-Ramírez M. E. Moran P. Rojas L. Portillo T. (2017). Human intestinal microbiota: interaction between parasites and the host immune response. *Arch Med. Res.* 48 690–700. 10.1016/j.arcmed.2017.11.015 29290328

[B59] Ras R. Huynh K. Desoky E. Badawy A. Widmer G. (2015). Perturbation of the intestinal microbiota of mice infected with *Cryptosporidium parvum*. *Int. J. Parasitol.* 45 567–573. 10.1016/j.ijpara.2015.03.005 25913477

[B60] Rohart F. Gautier B. Singh A. Lê Cao K.-A. (2017). mixOmics: an R package for ‘omics feature selection and multiple data integration. *PLoS Comput. Biol.* 13:e1005752 10.1371/journal.pcbi.1005752 PMC5687754 29099853

[B61] Schokker D. Zhang J. Zhang L. L. Vastenhouw S. A. Heilig H. G. Smidt H. (2014). Early-life environmental variation affects intestinal microbiota and immune development in new-born piglets. *PLoS One* 9:e100040 10.1371/journal.pone.0100040 PMC4062469 24941112

[B62] Schwab C. Gänzle M. (2011). Lactic acid bacteria fermentation of human milk oligosaccharide components, human milk oligosaccharides and galactooligosaccharides. *FEMS Microbiol. Lett.* 315 141–148. 10.1111/j.1574-6968.2010.02185.x 21175746

[B63] Shrestha A. Metzler-Zebeli B. U. Karembe H. Sperling D. Joachim A. (2020). The fecal microbiota evolves differently in piglets experimentally infected with *Cystoisospora suis* that are treated with toltrazuril. *Res. Square* 40 10.21203/rs.2.22641/v1

[B64] Stanley D. Hughes R. J. Moore R. J. (2014). Microbiota of the chicken gastrointestinal tract: influence on health, productivity and disease. *Appl. Microbiol. Biotechnol.* 98 4301–4310. 10.1007/s00253-014-5646-2 24643736

[B65] Stuart B. P. Lindsay D. S. Ernst J. V. Gosser H. S. (1980). *Isospora suis* enteritis in piglets. *Vet. Pathol.* 17 84–93. 7352366 10.1177/030098588001700109

[B66] Tito R. Y. Cypers H. Joossens M. Varkas G. Van Praet L. Glorieus E. (2017). Brief report: *Dialister* as a microbial marker of disease activity in spondyloarthritis. *Arthritis Rheum* 69 114–121. 10.1002/art.39802 27390077

[B67] Unterweger C. Schwarz L. Viehmann M. von Altrock A. Gerlach G. F. Waldmann K.-H. (2018). Treatment with probiotic bacteria does not diminish the impact of a *Cystoisospora suis* challenge in suckling piglets. *Front. Vet. Sci.* 5:313 10.3389/fvets.2018.00313 PMC6299013 30619896

[B68] Uzal F. A. McClane B. A. (2011). Recent progress in understanding the pathogenesis of *Clostridium perfringens* type C infections. *Vet. Microbiol.* 153 37–43. 10.1016/j.vetmic.2011.02.048 21420802 PMC3151542

[B69] Vandeputte D. Falony G. Vieira-Silva S. Tito R. Y. Joossens M. Raes J. (2016). Stool consistency is strongly associated with gut microbiota richness and composition, enterotypes and bacterial growth rates. *Gut* 65 57–62. 10.1136/gutjnl-2015-309618 26069274 PMC4717365

[B70] Vítovec J. Koudela B. (1990). Double alteration of the small intestine in conventional and gnotobiotic piglets experimentally infected with the coccidium *Isospora suis* (Apicomplexa, Eimeriidae). *Folia Parasitol.* 37 21–33. 2332196

[B71] Vítovec J. Koudela B. Kudweis M. Stěpánek J. Smíd B. Dvorák R. (1991). Pathogenesis of experimental combined infections with *Isospora suis* and Rotavirus in conventional and gnotobiotic piglets. *J. Vet. Med. B* 38 215–226. 10.1111/j.1439-0450.1991.tb00864.x 1858460

[B72] Wang X. Tsai T. Deng F. Wei X. Chai J. Knapp J. (2019). Longitudinal investigation of the swine gut microbiome from birth to market reveals stage and growth performance associated bacteria. *Microbiome* 7:109 10.1186/s40168-019-0721-7 PMC6664762 31362781

[B73] Worliczek H. L. Mundt H. C. Ruttkowski B. Joachim A. (2009). Age, not infection dose, determines the outcome of *Isospora suis* infections in suckling piglets. *Parasitol. Res.* 105(Suppl. 1) S157–S162. 10.1007/s00436-009-1507-9 19575237

[B74] Wu S.-B. Stanley D. Rodgers N. Swick R. A. Moore R. J. (2014). Two necrotic enteritis predisposing factors, dietary fishmeal and *Eimeria* infection, induce large changes in the caecal microbiota of broiler chickens. *Vet. Microbiol.* 169 188–197. 10.1016/j.vetmic.2014.01.007 24522272

[B75] Yang Q. Huang X. Zhao S. Sun W. Yan Z. Wang P. (2017). Structure and function of the fecal microbiota in diarrheic neonatal piglets. *Front. Microbiol.* 8:502 10.3389/fmicb.2017.00502 PMC5364137 28392784

[B76] Zhong X. Zhang Z. Wang S. Cao L. Zhou L. Sun A. (2019). Microbial-driven butyrate regulates jejunal homeostasis in piglets during the weaning stage. *Front. Microbiol.* 9:3335 10.3389/fmicb.2018.03335 PMC6345722 30713531

