# Supplementary figures and images for: Shifts in the Fecal Microbial Community of Cystoisospora suis Infected Piglets in Response to Toltrazuril
Source: Front Microbiol. 2020 May 19;11:983. doi: 10.3389/fmicb.2020.00983 (PMC7249887; doi:10.3389/fmicb.2020.00983)

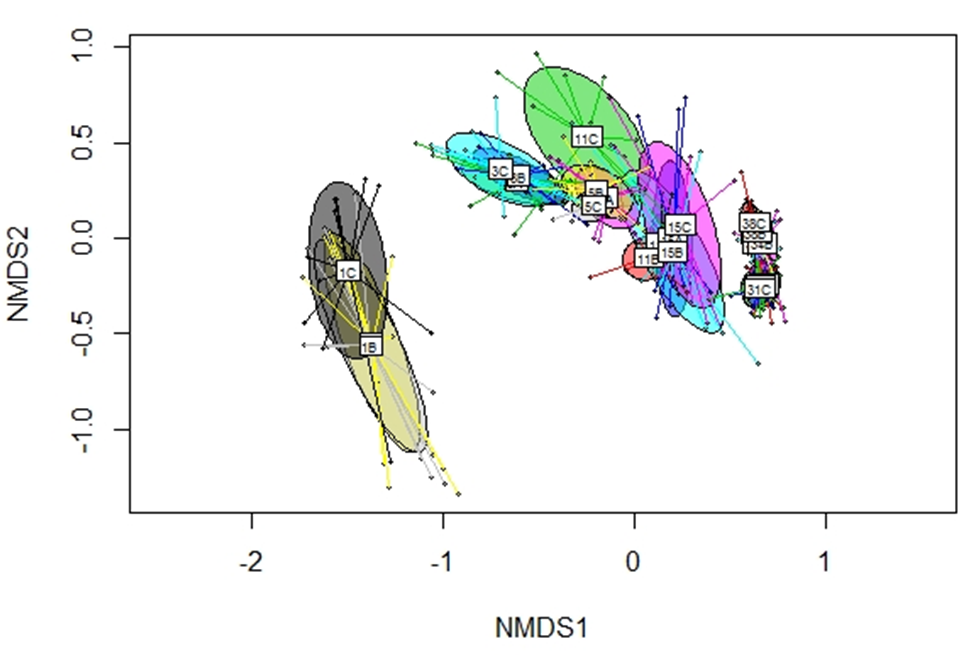

Supplement: FIGURE S1 — Mean fecal scores (all groups) and oocyst excretion (ln of oocysts per gram [OPG]; control only) during the study period. All groups: infection with C. suis on the first day of life. Groups: Parenteral Toltra (treatment with parenteral toltrazuril on the second day of life), Oral Toltra (treatment with oral toltrazuril on the fourth day of life), Control: no treatment. [file Image_1.TIF]

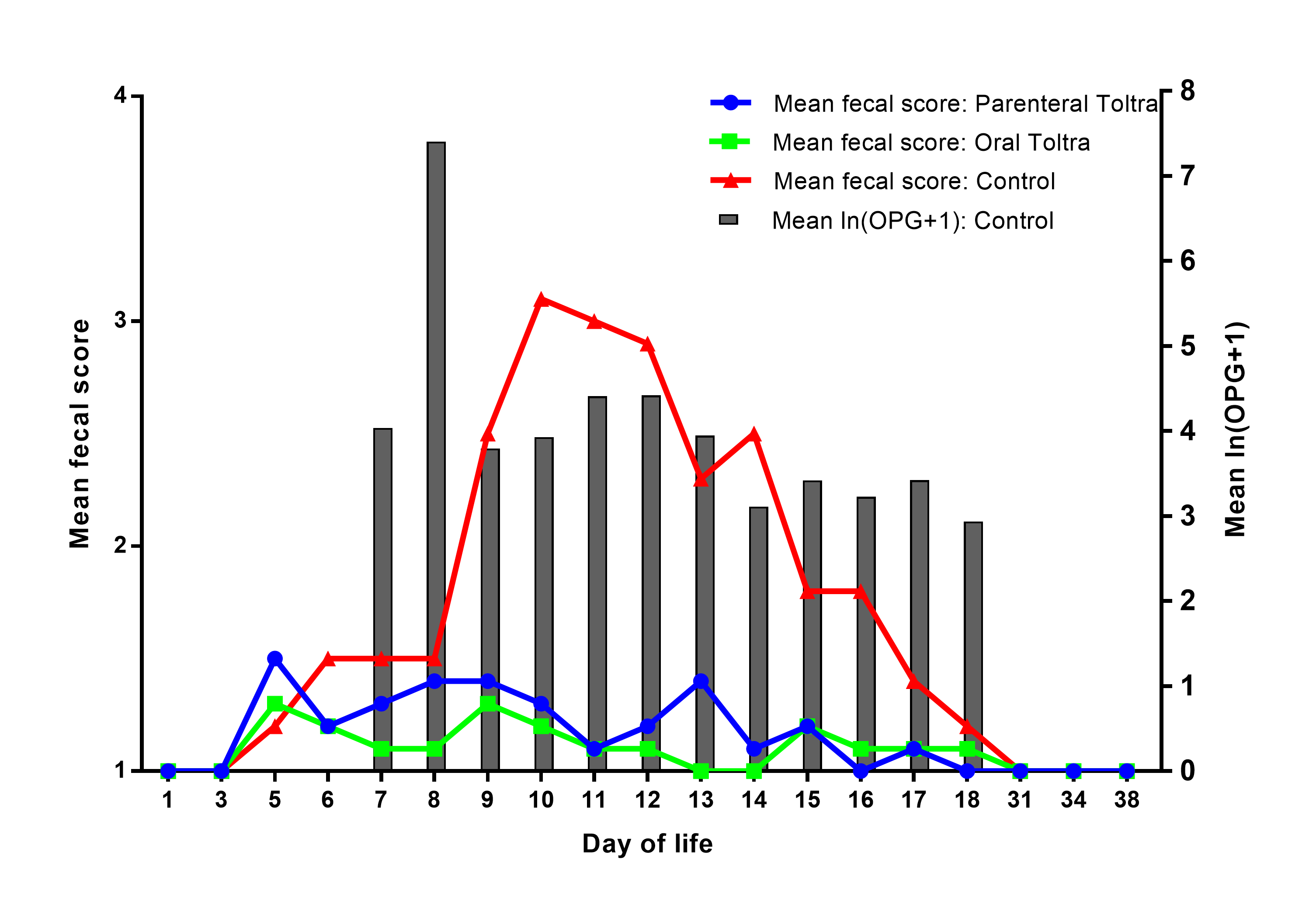

Supplement: FIGURE S2 — Non-metric multidimensional scaling plot of pairwise Bray-Curtis dissimilarities between bacterial communities at genus level in feces of suckling and weaned piglets (>0.01% relative abundance) showing the treatment effects on all sampling time points (day of life: 1, 3, 5, 11, 15, 31, 34, and 38; stress: 0.1067) between different groups (group A = parenteral toltrazuril, group B = oral toltrazuril, group C = untreated control group). [file Image_2.TIF]
