# Supplementary material for: Shifts in the Fecal Microbial Community of Cystoisospora suis Infected Piglets in Response to Toltrazuril
Source: Front Microbiol. 2020 May 19;11:983. doi: 10.3389/fmicb.2020.00983 (PMC7249887; doi:10.3389/fmicb.2020.00983)
Supplement: TABLE S1 — Comparison of groups: Oocyst excretion, diarrhea, body weight gain and concomitant infections. OpG: oocysts per gram of feces, dol: day of life, g: gram. [file Data_Sheet_1.docx]

**Supplementary table S1:** Comparison of groups: Oocyst excretion, diarrhea, body weight gain and concomitant infections. OpG: oocysts per gram of feces, dol: day of life, g: gram.

| **Parameters** | **Parenteral Toltrazuril** | **Oral Toltrazuril** | **Control** |
| --- | --- | --- | --- |
| No. of included piglets | 13 | 13 | 11 |
| Total number of samples; 14 days sampling | 179 | 180 | 154 |
| **Oocyst excretion** | | | |
| No. of piglets positive in McMaster (%) | 0 (0) | 0 (0) | 11 (100) |
| No. of excretion days (%) | 0 (0) | 0 (0) | 67 (43.5%) |
| Mean area under the curve for OpG | 0 | 0 | 17,162 |
| **Fecal Consistency and diarrhea** | | | |
| No. of piglets with diarrhea (%) | 1 (7.7) | 0(0) | 11 (100) |
| No. piglets with watery diarrhea (%) | 1 (7.7) | 0 (0) | 8 (72.7) |
| No. of days with diarrhea (%) | 5 (2.8) | 0 (0) | 40 (26.0) |
| No. of days with watery diarrhea (%) | 2 (1.1) | 0 (0) | 16 (10.4) |
| Mean area under the curve for fecal score | 16.0 | 14.4 | 26.7 |
| **Body weight development** | | | |
| Mean body weight gain (g) dol 1- dol 29 | 5701.7 | 5484.6 | 4894.5 |
| Mean daily body weight gain (g) dol 1- dol 29 | 203.6 | 195.9 | 174.8 |
| Mean daily body weight gain (g) dol 8- dol 15 | 1486.2 | 1446.9 | 447.3 |
| **Concomitant infections (pooled samples on dol 5)** |  | | |
| *E. coli* | +++ | +++ | +++ |
| *Cl. perfringens* type A (*Cp*A) | ++ | ++ | ++ |
| Rotavirus | - | - | - |
| Corona virus | - | - | - |

**Supplementary table S2.** Total bacterial 16S rRNA gene copy numbers by treatments groups and days of life. Different superscript letters mark significant differences at *P* <0.05. SEM: standard error of the mean.

| **Total bacteria (log10 gene copies/g feces)** | **Toltrazuril parenteral** | **Toltrazuril oral** | **Control** | **SEM** | **Treatment effect** |
| --- | --- | --- | --- | --- | --- |
| Day 1 of life (day of infection) | 8.3^ab^ | 7.9^b^ | 8.6^a^ | 0.193 | **0.039** |
| Day 3 of life (day of treatment) | 8.6^b^ | 8.9^ab^ | 9.1^a^ | 0.138 | **0.075** |
| Day 5 of life | 9.0 | 9.0 | 9.0 | 0.105 | 0.998 |
| Day 11 of life | 9.0 | 9.1 | 9.1 | 0.112 | 0.810 |
| Day 15 of life | 8.6a^b^ | 7.8^b^ | 8.9^a^ | 0.284 | **0.033** |
| Day 31 of life | 8.8 | 9.0 | 9.0 | 0.129 | 0.307 |
| Day 34 of life | 9.0 | 9.1 | 9.1 | 0.018 | 0.590 |
| Day 38 of life | 9.1 | 9.1 | 9.1 | 0.010 | 0.997 |

**Supplementary table S3.** Differences in the relative abundances (> 0.01% of all reads) of bacterial phyla in feces of piglets on day 1, 3, 5, 11, 15, 31, 34 and 38 of life. Different superscript letters mark significant differences at *P*<0.05. SEM: standard error of the mean.

| **Phylum** | **Parenteral toltrazuril** | **Oral toltrazuril** | **Control** | **SEM** | **Treatment effect** |
| --- | --- | --- | --- | --- | --- |
| Day 1 of life |  |  |  |  |  |
| p__*Firmicutes* | 25.00 | 23.03 | 17.85 | 4.055 | 0.457 |
| p__*Bacteroidetes* | 6.07 | 9.70 | 9.51 | 3.104 | 0.644 |
| p__*Fusobacteria* | 8.90 | 11.13 | 15.35 | 3.598 | 0.455 |
| p__*Proteobacteria* | 57.77 | 53.36 | 55.89 | 7.018 | 0.903 |
| p__*Actinobacteria* | 2.20 | 2.73 | 1.30 | 0.947 | 0.582 |
| Day 3 of life |  |  |  |  |  |
| p__*Firmicutes* | 20.05 | 15.50 | 17.97 | 1.659 | 0.155 |
| p__*Bacteroidetes* | 33.31 | 33.75 | 34.48 | 1.603 | 0.870 |
| p__*Fusobacteria* | 35.70 | 40.94 | 39.17 | 1.986 | 0.165 |
| p__*Proteobacteria* | 10.28 | 9.17 | 7.74 | 1.575 | 0.517 |
| p__*Actinobacteria* | 0.65 | 0.64 | 0.62 | 0.203 | 0.992 |
| p__*Spirochaetes* | 0 | 0.01 | 0.02 | 0.009 | 0.442 |
| Day 5 of life |  | |  |  |  |
| p__*Firmicutes* | 35.39 | 35.39 | 37.90 | 3.258 | 0.852 |
| p__*Bacteroidetes* | 38.50^a^ | 35.07^a^ | 28.60^b^ | 2.054 | **0.011** |
| p__*Fusobacteria* | 19.26 | 23.39 | 25.41 | 3.082 | 0.378 |
| p__*Proteobacteria* | 3.52 | 4.78 | 4.85 | 1.061 | 0.636 |
| p__*Actinobacteria* | 2.28 | 2.45 | 2.42 | 0.656 | 0.319 |
| p__*Spirochaetes* | 0.01 | 0.05 | 0 | 0.021 | 0.164 |
| p__*Lentisphaerae* | 0.02 | 0.02 | 0.01 | 0.009 | 0.827 |
| Day 11 of life |  | |  |  |  |
| p__*Firmicutes* | 59.17^a^ | 59.06^a^ | 34.29^b^ | 4.714 | **<0.001** |
| p__*Bacteroidetes* | 27.73 | 25.08 | 26.64 | 3.730 | 0.886 |
| p__*Fusobacteria* | 7.96^b^ | 5.54^b^ | 27.83^a^ | 3.544 | **<0.001** |
| p__*Proteobacteria* | 2.62^b^ | 6.88^a^ | 9.84^a^ | 1.386 | **0.003** |
| p__*Actinobacteria* | 1.14 | 1.88 | 0.77 | 0.353 | 0.105 |
| p__*Synergistetes* | 0.09^b^ | 0.61^a^ | 0.04^b^ | 0.154 | **0.029** |
| p__*Spirochaetes* | 0.27 | 0.21 | 0.18 | 0.110 | 0.830 |
| p__*Lentisphaerae* | 0.14^b^ | 0.23^a^ | 0.05^b^ | 0.040 | **0.016** |
| p__*Euryarchaeota* | 0.10^a^ | 0.11^a^ | 0.01^b^ | 0.031 | **0.044** |
| Day 15 of life |  |  |  |  |  |
| p__*Firmicutes* | 56.41 | 56.64 | 54.75 | 5.214 | 0.965 |
| p__*Bacteroidetes* | 26.72 | 21.04 | 26.85 | 2.789 | 0.262 |
| p__*Fusobacteria* | 5.37 | 9.52 | 4.76 | 3.304 | 0.555 |
| p__*Proteobacteria* | 5.55 | 6.67 | 7.27 | 1.423 | 0.679 |
| p__*Actinobacteria* | 1.89 | 1.64 | 1.17 | 0.507 | 0.612 |
| p__*Synergistetes* | 2.71 | 0.42 | 1.94 | 1.037 | 0.279 |
| p__*Spirochaetes* | 0.55 | 2.22 | 1.99 | 0.866 | 0.313 |
| p__*Lentisphaerae* | 0.31 | 0.15 | 0.23 | 0.122 | 0.637 |
| p__*Euryarchaeota* | 0.26 | 0.63 | 0.31 | 0.162 | 0.223 |
| Day 31 of life |  |  |  |  |  |
| p__*Firmicutes* | 63.97 | 62.33 | 66.95 | 2.530 | 0.442 |
| p__*Bacteroidetes* | 24.65 | 26.06 | 21.58 | 2.472 | 0.442 |
| p__*Fusobacteria* | 0.02 | 0.01 | 0.03 | 0.013 | 0.624 |
| p__*Proteobacteria* | 2.06 | 1.95 | 2.80 | 0.430 | 0.343 |
| p__*Actinobacteria* | 1.85 | 1.17 | 1.52 | 0.393 | 0.462 |
| p__*Synergistetes* | 0.18 | 0.17 | 0.15 | 0.072 | 0.951 |
| p__*Spirochaetes* | 2.25 | 3.47 | 2.54 | 0.571 | 0.280 |
| p__*Lentisphaerae* | 0.07 | 0.11 | 0.12 | 0.037 | 0.694 |
| p__*Euryarchaeota* | 0.69 | 1.18 | 0.77 | 0.248 | 0.328 |
| p__*Chlamydiae* | 0.75 | 0.13 | 0.54 | 0.291 | 0.301 |
| Day 34 of life |  |  |  |  |  |
| p__*Firmicutes* | 61.75 | 62.70 | 62.69 | 1.342 | 0.846 |
| p__*Bacteroidetes* | 30.27 | 29.79 | 32.21 | 1.595 | 0.543 |
| p__*Proteobacteria* | 2.31 | 1.89 | 1.23 | 0.627 | 0.498 |
| p__*Actinobacteria* | 1.31 | 1.63 | 1.12 | 0.258 | 0.373 |
| p__*Synergistetes* | 0.01 | 0.01 | 0.02 | 0.004 | 0.273 |
| p__*Spirochaetes* | 1.77 | 1.35 | 0.65 | 0.402 | 0.171 |
| p__*Euryarchaeota* | 0.27 | 0.20 | 0.15 | 0.055 | 0.336 |
| p__*Chlamydiae* | 0.92 | 0.63 | 0.63 | 0.218 | 0.561 |
| Day 38 of life |  |  |  |  |  |
| p__*Firmicutes* | 59.75 | 61.55 | 60.98 | 1.222 | 0.565 |
| p__*Bacteroidetes* | 34.02 | 31.83 | 33.41 | 1.133 | 0.361 |
| p__*Proteobacteria* | 2.29 | 1.90 | 1.97 | 0.399 | 0.764 |
| p__*Actinobacteria* | 1.33 | 1.74 | 1.50 | 0.236 | 0.470 |
| p__*Spirochaetes* | 0.93 | 0.84 | 0.74 | 0.240 | 0.860 |
| p__*Euryarchaeota* | 0.10 | 0.10 | 0.06 | 0.038 | 0.679 |
| p__*Chlamydiae* | 0.25 | 0.39 | 0.28 | 0.077 | 0.361 |

**Supplementary table S4**. Differences in the relative abundances (> 0.01% of all reads) of bacterial genera in feces of piglets on day 1, 3, 5, 11, 15, 31, 34 and 38 of life. Different superscript letters mark significant differences at *P*<0.05. SEM: standard error of the mean.

| **Genus** | **Parenteral toltrazuril** | **Oral toltrazuril** | **Control** | **SEM** | **Treatment effect** |
| --- | --- | --- | --- | --- | --- |
| Day 1 of life |  |  |  |  |  |
| g__*Fusobacterium* | 8.96 | 11.18 | 15.41 | 3.619 | 0.459 |
| g__*Prevotella* | 0.05 | 0.03 | 0.23 | 0.088 | 0.247 |
| g__*Bacteroides* | 1.29 | 6.16 | 7.33 | 2.203 | 0.134 |
| g__*Lactobacillus* | 6.45 | 7.23 | 3.86 | 1.924 | 0.459 |
| g__*Escherichia* | 44.51 | 38.91 | 37.22 | 8.009 | 0.796 |
| f__*S24-7*; g_ | 0.01 | 0.01 | 0 | 0.006 | 0.322 |
| g__*Clostridium* | 13.67 | 10.11 | 7.16 | 3.113 | 0.349 |
| f__*Clostridiaceae*; g_ | 0.17 | 0.18 | 0.09 | 0.037 | 0.213 |
| g__*Phascolarctobacterium* | 0.01 | 0.02 | 0.02 | 0.010 | 0.777 |
| g__*SMB53* | 0.14 | 0.10 | 0.24 | 0.085 | 0.499 |
| g__*Roseburia* | 0.03 | 0.01 | 0.01 | 0.012 | 0.553 |
| g__*Klebsiella* | 7.32 | 8.08 | 11.86 | 1.580 | 0.120 |
| g__*Megasphaera* | 0 | 0.07 | 0.28 | 0.131 | 0.311 |
| g__*Streptococcus* | 0.87 | 1.05 | 1.19 | 0.275 | 0.718 |
| g__*Veillonella* | 0.74 | 0.81 | 2.03 | 0.605 | 0.270 |
| g__*Turicibacter* | 0.03 | 0.06 | 0.02 | 0.022 | 0.453 |
| g__*Pasteurella* | 0.97 | 0.85 | 1.02 | 0.414 | 0.955 |
| g__*Sutterella* | 0.09^b^ | 0.23^b^ | 1.49^a^ | 0.343 | **0.015** |
| g__*Mitsuokella* | 0.04 | 0.03 | 0.12 | 0.057 | 0.539 |
| g__*Wautersiella* | 3.91 | 2.92 | 1.55 | 1.791 | 0.655 |
| g__*Actinobacillus* | 0.72 | 0.68 | 1.03 | 0.401 | 0.812 |
| g__*Corynebacterium* | 1.75 | 2.18 | 1.06 | 0.744 | 0.585 |
| f__*Peptostreptococcaceae*; g_ | 0.01 | 0.04 | 0.07 | 0.038 | 0.501 |
| g__*Bifidobacterium* | 0.10 | 0.15 | 0.06 | 0.043 | 0.350 |
| g__*Acidaminococcus* | 0 | 0.02 | 0.19 | 0.073 | 0.142 |
| g__*Acetobacter* | 2.01 | 2.09 | 1.07 | 1.102 | 0.778 |
| Day 3 of life |  |  |  |  |  |
| g__*Fusobacterium* | 35.75 | 40.99 | 39.23 | 1.982 | 0.164 |
| g__*Prevotella* | 7.00 | 4.33 | 4.89 | 1.823 | 0.536 |
| g__*Bacteroides* | 23.63 | 26.40 | 27.39 | 1.936 | 0.355 |
| g__*Lactobacillus* | 7.85 | 6.45 | 7.05 | 1.235 | 0.713 |
| g__*Escherichia* | 6.37 | 5.40 | 4.35 | 1.620 | 0.673 |
| f__*Lachnospiraceae*; g_ | 0.51 | 0.32 | 0.58 | 0.209 | 0.666 |
| f__*Ruminococcaceae*; g_ | 0.08 | 0.24 | 0.05 | 0.099 | 0.373 |
| f__*S24-7*; g_ | 0.70 | 0.47 | 0.41 | 0.204 | 0.570 |
| g__*Oscillospira* | 0.06 | 0.08 | 0.03 | 0.021 | 0.259 |
| g__*Clostridium* | 1.62 | 0.91 | 1.47 | 0.512 | 0.586 |
| f__*Clostridiaceae*; g_ | 0.04 | 0 | 0.09 | 0.039 | 0.350 |
| g__*Ruminococcus* | 0.10 | 0.17 | 0.22 | 0.077 | 0.524 |
| g__*Phascolarctobacterium* | 0.18 | 0.15 | 0.35 | 0.092 | 0.273 |
| g__*SMB53* | 0.12 | 0.07 | 0.08 | 0.038 | 0.553 |
| o__*Clostridiales*; f_; g_ | 0.00 | 0.00 | 0.20 | 0.105 | 0.282 |
| g__*Roseburia* | 0.46 | 0.12 | 0.14 | 0.194 | 0.374 |
| g__*Blautia* | 0.23 | 0.19 | 0.58 | 0.225 | 0.420 |
| g__*Klebsiella* | 0.22 | 0.16 | 0.16 | 0.108 | 0.905 |
| g__*Megasphaera* | 0.48 | 0.28 | 0.23 | 0.265 | 0.763 |
| g__*Anaerovibrio* | 0.10 | 0.01 | 0.22 | 0.125 | 0.504 |
| o__*Bacteroidales*; f_; g_ | 0.01 | 0.02 | 0.03 | 0.023 | 0.863 |
| g__*Coprococcus* | 0.08 | 0.03 | 0.04 | 0.028 | 0.368 |
| g__[*Prevotella*] | 0.58 | 0.68 | 0.84 | 0.375 | 0.881 |
| g__[*Ruminococcus*] | 0.13 | 0.27 | 0.08 | 0.076 | 0.209 |
| g__[*Eubacterium*] | 0.32 | 0.43 | 0.34 | 0.105 | 0.715 |
| g__*Dorea* | 1.74 | 0.96 | 1.18 | 0.303 | 0.168 |
| g__*Streptococcus* | 1.07 | 0.68 | 1.00 | 0.131 | 0.092 |
| g__*Faecalibacterium* | 0.14 | 0.36 | 0.17 | 0.108 | 0.296 |
| g__*Veillonella* | 2.09^a^ | 1.16^b^ | 1.64^ab^ | 0.246 | **0.035** |
| f__[*Mogibacteriaceae*]; g_ | 0.01 | 0.05 | 0.10 | 0.051 | 0.432 |
| g__*Butyricimonas* | 1.26 | 1.60 | 0.78 | 0.359 | 0.284 |
| g__*Collinsella* | 0.11 | 0.09 | 0.07 | 0.034 | 0.730 |
| g__*Campylobacter* | 0.24 | 0.46 | 0.35 | 0.146 | 0.559 |
| g__*Clostridium* | 0.59 | 0.57 | 0.17 | 0.201 | 0.267 |
| f__*Coriobacteriaceae*; g_ | 0.15 | 0.09 | 0.12 | 0.077 | 0.874 |
| g__*Pasteurella* | 1.37 | 0.90 | 1.09 | 0.353 | 0.623 |
| g__*Sutterella* | 0.47 | 0.53 | 0.41 | 0.089 | 0.652 |
| f__*Rikenellaceae*; g_ | 0.10 | 0.26 | 0.13 | 0.064 | 0.208 |
| g__*Mitsuokella* | 0.05 | 0.11 | 0.29 | 0.201 | 0.673 |
| g__*Sarcina* | 0.03 | 0.01 | 0 | 0.012 | 0.306 |
| g__*Actinobacillus* | 1.34 | 0.61 | 1.01 | 0.369 | 0.369 |
| g__*Desulfovibrio* | 0.21 | 0.23 | 0.18 | 0.043 | 0.759 |
| g__*Butyricicoccus* | 0.14 | 0.18 | 0.13 | 0.036 | 0.582 |
| g__*Parabacteroides* | 0.05 | 0.04 | 0.01 | 0.026 | 0.404 |
| g__*Corynebacterium* | 0.04^a^ | 0.01^b^ | 0.01^b^ | 0.009 | **0.023** |
| g__*p-75-a5* | 0.02 | 0.09 | 0.01 | 0.031 | 0.142 |
| g__*Bulleidia* | 0.02 | 0.04 | 0.06 | 0.023 | 0.464 |
| g__*Peptostreptococcus* | 0.88 | 0.73 | 0.68 | 0.174 | 0.696 |
| g__*Bifidobacterium* | 0.13 | 0.16 | 0.18 | 0.111 | 0.935 |
| g__*Acidaminococcus* | 0.11 | 0.06 | 0.08 | 0.095 | 0.929 |
| g__*CF231* | 0.02 | 0.00 | 0.05 | 0.023 | 0.210 |
| Day 5 of life |  |  |  |  |  |
| g__*Fusobacterium* | 20.28 | 24.18 | 25.22 | 3.103 | 0.498 |
| g__*Prevotella* | 4.86 | 4.90 | 2.47 | 1.160 | 0.315 |
| g__*Bacteroides* | 24.16 | 23.50 | 20.24 | 2.596 | 0.587 |
| g__*Lactobacillus* | 10.65 | 13.23 | 15.09 | 2.249 | 0.412 |
| g__*Escherichia* | 0.97 | 1.57 | 1.66 | 0.548 | 0.620 |
| f__*Lachnospiraceae*; g_ | 3.76 | 2.77 | 4.64 | 1.095 | 0.503 |
| f__*Ruminococcaceae*; g_ | 0.89 | 0.72 | 0.57 | 0.318 | 0.801 |
| f__*S24-7*; g_ | 6.55 | 3.30 | 4.16 | 1.860 | 0.398 |
| g__*Oscillospira* | 1.74 | 1.55 | 1.04 | 0.553 | 0.696 |
| g__*Clostridium* | 2.17 | 1.07 | 1.27 | 0.745 | 0.505 |
| f__*Clostridiaceae*; g_ | 0.17 | 0.12 | 0.06 | 0.089 | 0.742 |
| g__*Ruminococcus* | 1.73 | 0.68 | 1.08 | 0.438 | 0.193 |
| g__*Phascolarctobacterium* | 1.46 | 0.71 | 0.40 | 0.358 | 0.124 |
| g__*SMB53* | 0.22 | 0.28 | 0.23 | 0.071 | 0.736 |
| o__*Clostridiales*; f_; g_ | 0.37 | 0.41 | 0.19 | 0.133 | 0.522 |
| g__*Roseburia* | 0.18 | 0.10 | 0.07 | 0.044 | 0.207 |
| g__*Blautia* | 0.77 | 0.57 | 1.93 | 0.382 | 0.060 |
| g__*Megasphaera* | 0.26 | 0.27 | 0.33 | 0.105 | 0.916 |
| f__*Christensenellaceae*; g_ | 0.02 | 0.01 | 0.01 | 0.006 | 0.242 |
| o__*Bacteroidales*; f_; g_ | 0.11 | 0.23 | 0.11 | 0.085 | 0.458 |
| g__*Coprococcus* | 0.37^a^ | 0.11^b^ | 0.13^b^ | 0.070 | **0.019** |
| g__[*Prevotella*] | 0.69 | 1.38 | 0.30 | 0.569 | 0.403 |
| g__[*Ruminococcus*] | 1.17 | 0.79 | 0.57 | 0.300 | 0.392 |
| g__[*Eubacterium*] | 1.51 | 0.96 | 1.66 | 0.319 | 0.257 |
| g__*Dorea* | 1.54 | 1.44 | 1.20 | 0.360 | 0.816 |
| g__*Streptococcus* | 0.83 | 1.02 | 1.39 | 0.218 | 0.259 |
| g__*Faecalibacterium* | 0.49 | 0.42 | 0.39 | 0.169 | 0.907 |
| g__*Succinivibrio* | 0.01 | 0.02 | 0.03 | 0.016 | 0.685 |
| g__*Veillonella* | 0.60 | 0.55 | 0.68 | 0.166 | 0.870 |
| f__[*Mogibacteriaceae*]; g_ | 0.41 | 0.37 | 0.53 | 0.174 | 0.830 |
| g__*Butyricimonas* | 1.44 | 1.19 | 1.24 | 0.307 | 0.808 |
| g__*Collinsella* | 0.36 | 0.18 | 0.37 | 0.169 | 0.648 |
| g__*Turicibacter* | 0.05 | 0.03 | 0.01 | 0.024 | 0.638 |
| g__*Campylobacter* | 0.32 | 0.47 | 1.29 | 0.482 | 0.387 |
| g__*Clostridium* | 1.25 | 1.63 | 1.08 | 0.360 | 0.534 |
| f__*Coriobacteriaceae*; g_ | 0.90 | 0.76 | 0.67 | 0.296 | 0.877 |
| g__*Pasteurella* | 0.30 | 0.57 | 0.34 | 0.193 | 0.522 |
| g__*Sutterella* | 0.30 | 0.27 | 0.16 | 0.088 | 0.585 |
| f__*Rikenellaceae*; g_ | 0.52 | 0.66 | 0.38 | 0.176 | 0.568 |
| g__*Mitsuokella* | 0.01 | 0.10 | 0.05 | 0.030 | 0.088 |
| g__*Actinobacillus* | 0.06 | 0.57 | 0.45 | 0.276 | 0.350 |
| g__*Desulfovibrio* | 0.48 | 0.58 | 0.50 | 0.093 | 0.716 |
| g__*Butyricicoccus* | 0.51 | 0.30 | 0.40 | 0.125 | 0.436 |
| g__*Parabacteroides* | 0.17 | 0.31 | 0.21 | 0.113 | 0.632 |
| g__*Corynebacterium* | 0.11 | 0.03 | 0.04 | 0.059 | 0.552 |
| g__*p-75-a5* | 0.19 | 0.83 | 0.49 | 0.269 | 0.203 |
| g__*Bulleidia* | 0.12 | 0.26 | 0.33 | 0.136 | 0.568 |
| f__*Peptostreptococcaceae*; g_ | 0.06 | 0.05 | 0.05 | 0.045 | 0.992 |
| g__*Peptostreptococcus* | 1.04 | 0.75 | 0.93 | 0.242 | 0.652 |
| g__*Bifidobacterium* | 0.15^b^ | 0.70^a^ | 0.37^ab^ | 0.137 | **0.016** |
| f_*Erysipelaceae*; g_ | 0.12^b^ | 0.18^ab^ | 0.33^a^ | 0.057 | **0.050** |
| g__*Acidaminococcus* | 0.04 | 0.02 | 0.01 | 0.012 | 0.192 |
| f__*Prevotellaceae*; g_ | 0.23 | 0.01 | 0.17 | 0.097 | 0.200 |
| g__*CF231* | 0.17 | 0.14 | 0.05 | 0.107 | 0.754 |
| Day 11 of life |  |  |  |  |  |
| g__*Fusobacterium* | 7.75^b^ | 5.90^b^ | 26.88^a^ | 3.012 | **<0.001** |
| g__*Prevotella* | 3.82 | 2.52 | 8.78 | 2.146 | 0.115 |
| g__*Bacteroides* | 10.72 | 14.03 | 14.36 | 2.906 | 0.613 |
| g__*Lactobacillus* | 16.92 | 16.71 | 8.49 | 3.193 | 0.119 |
| g_*Escherichia* | 1.16^b^ | 4.69^a^ | 1.73^b^ | 0.923 | **0.029** |
| f__*Lachnospiraceae*; g_ | 5.76^a^ | 5.44^a^ | 1.27^b^ | 0.819 | **<0.001** |
| f_*Ruminococcaceae*; g_ | 3.92^a^ | 3.62^a^ | 0.97^b^ | 0.845 | **0.036** |
| f__*S24-7*; g_ | 6.52^a^ | 4.10^a^ | 0.81^b^ | 0.899 | **<0.001** |
| g__*Oscillospira* | 7.86^a^ | 6.91^a^ | 1.55^b^ | 1.099 | **<0.001** |
| g__*Clostridium* | 1.33 | 1.25 | 0.26 | 0.404 | 0.129 |
| f__*Clostridiaceae*; g_ | 0.97 | 0.60 | 0.09 | 0.333 | 0.173 |
| g_*Ruminococcus* | 4.23^a^ | 4.32^a^ | 1.22^b^ | 0.905 | **0.034** |
| g__*Phascolarctobacterium* | 1.55 | 1.54 | 0.95 | 0.286 | 0.249 |
| g_*SMB53* | 1.69^a^ | 1.40^a^ | 0.23^b^ | 0.368 | **0.020** |
| o__*Clostridiales*; f_; g_ | 1.63 | 1.21 | 2.76 | 0.520 | 0.113 |
| g__*Roseburia* | 0.23 | 0.23 | 0.31 | 0.120 | 0.867 |
| g_*Blautia* | 1.50^a^ | 0.59^b^ | 0.46^b^ | 0.291 | **0.030** |
| g__*Klebsiella* | 0.02 | 0.04 | 0 | 0.014 | 0.260 |
| g__*Megasphaera* | 0.60 | 0.61 | 3.19 | 1.023 | 0.134 |
| g_*Anaerovibrio* | 0.080^b^ | 0.056^b^ | 3.93^a^ | 1.077 | **0.023** |
| f_*Christensenellaceae*; g_ | 1.56^a^ | 1.30^a^ | 0.19^b^ | 0.403 | **0.050** |
| o_*Bacteroidales*; f_; g_ | 0.84^a^ | 0.50^ab^ | 0.055^b^ | 0.213 | **0.042** |
| g__*Coprococcus* | 0.21 | 0.18 | 0.08 | 0.068 | 0.382 |
| g__[*Prevotella*] | 0.53 | 0.45 | 0.90 | 0.345 | 0.619 |
| g_[*Ruminococcus*] | 1.85^a^ | 1.89^a^ | 0.53^b^ | 0.326 | **0.008** |
| g__[*Eubacterium*] | 2.24^a^ | 2.35^a^ | 0.27^b^ | 0.458 | **0.005** |
| g__*Dorea* | 0.88 | 1.04 | 0.81 | 0.258 | 0.826 |
| g__*Treponema* | 0.20 | 0.15 | 0.13 | 0.083 | 0.797 |
| g__*Streptococcus* | 0.81 | 1.09 | 2.08 | 0.401 | 0.075 |
| g__*Faecalibacterium* | 0.54 | 0.64 | 0.04 | 0.192 | 0.080 |
| g__*Succinivibrio* | 0.11 | 0.52 | 1.81 | 0.544 | 0.080 |
| g__*Veillonella* | 0.36 | 0.29 | 0.88 | 0.181 | 0.058 |
| f_*Mogibacteriaceae*; g_ | 0.46^b^ | 0.75^a^ | 0.088^a^ | 0.147 | **0.014** |
| g__*Butyricimonas* | 1.12 | 1.11 | 0.36 | 0.220 | **0.030** |
| g__*Collinsella* | 0.54 | 0.21 | 0.56 | 0.209 | 0.439 |
| g__*Turicibacter* | 0.53 | 0.53 | 0.06 | 0.160 | 0.072 |
| g__*Campylobacter* | 0.44 | 0 | 3.57 | 1.118 | 0.065 |
| g__*Clostridium* | 0.40 | 0.68 | 0.55 | 0.198 | 0.615 |
| f__*Coriobacteriaceae*; g_ | 0.21 | 0.71 | 0.03 | 0.195 | 0.060 |
| g__*Pasteurella* | 0.20 | 0.29 | 0.13 | 0.117 | 0.639 |
| g__*Sutterella* | 0.32^b^ | 0.11^b^ | 2.81^a^ | 0.488 | **0.001** |
| f__*Rikenellaceae*; g_ | 1.30^a^ | 1.95^a^ | 0.51^b^ | 0.322 | 0.**015** |
| g__*Mitsuokella* | 0.06 | 0.47 | 0.47 | 0.256 | 0.421 |
| g__*Sarcina* | 0 | 0.01 | 0.36 | 0.187 | 0.324 |
| g__*Actinobacillus* | 0.11 | 0.07 | 0.25 | 0.061 | 0.104 |
| f__*RFP12*; g_ | 0.48 | 0.02 | 0.03 | 0.331 | 0.536 |
| g__*Desulfovibrio* | 0.69^a^ | 0.76^a^ | 0.17^b^ | 0.106 | **0.001** |
| g__*Butyricicoccus* | 0.10 | 0.10 | 0.09 | 0.036 | 0.977 |
| g__*Parabacteroides* | 0.53 | 0.61 | 0.64 | 0.196 | 0.913 |
| g__*Corynebacterium* | 0.09 | 0.05 | 0.02 | 0.020 | 0.073 |
| g__*p-75-a5* | 0.63 | 0.64 | 0.06 | 0.209 | 0.103 |
| g__*Bulleidia* | 0.18 | 0.22 | 0.11 | 0.074 | 0.562 |
| f__*Peptostreptococcaceae*; g_ | 0.15 | 0.07 | 0.03 | 0.050 | 0.257 |
| g__*Peptostreptococcus* | 0.03 | 0.10 | 0.32 | 0.121 | 0.218 |
| g_*Bifidobacterium* | 0.010^b^ | 0.44^a^ | 0.091^b^ | 0.079 | **0.007** |
| g__*Acidaminococcus* | 0.13 | 0.13 | 0.23 | 0.093 | 0.654 |
| f__*RF16*; g_ | 0.02 | 0.04 | 0 | 0.016 | 0.226 |
| f__*Prevotellaceae*; g_ | 0.59 | 0.46 | 0.06 | 0.198 | 0.149 |
| f__*Pirellulaceae*; g | 0.17 | 0.23 | 0.06 | 0.075 | 0.266 |
| g__*CF231* | 0.03 | 0.05 | 0.37 | 0.196 | 0.392 |
| Day 15 of life |  |  |  |  |  |
| g__*Fusobacterium* | 5.38 | 9.60 | 4.77 | 3.303 | 0.546 |
| g__*Prevotella* | 6.58 | 4.40 | 8.37 | 2.188 | 0.475 |
| g__*Bacteroides* | 10.28 | 7.61 | 11.51 | 2.265 | 0.490 |
| g__*Lactobacillus* | 10.32 | 9.79 | 6.65 | 1.768 | 0.319 |
| g__*Escherichia* | 3.30 | 4.14 | 3.65 | 1.207 | 0.878 |
| f__*Lachnospiraceae*; g_ | 3.55 | 3.82 | 6.56 | 1.375 | 0.270 |
| f__*Ruminococcaceae*; g_ | 3.37 | 3.64 | 2.62 | 0.841 | 0.703 |
| f__*S24-7*; g_ | 4.61 | 3.64 | 2.17 | 1.031 | 0.261 |
| g__*Oscillospira* | 6.68 | 8.19 | 5.43 | 1.608 | 0.514 |
| g__*Clostridium* | 1.15 | 2.10 | 0.83 | 0.904 | 0.608 |
| f__*Clostridiaceae*; g_ | 0.58 | 1.21 | 0.24 | 0.500 | 0.416 |
| g__*Ruminococcus* | 4.48 | 2.69 | 5.19 | 1.415 | 0.464 |
| g__*Phascolarctobacterium* | 2.48 | 2.41 | 1.91 | 0.472 | 0.667 |
| g__*SMB53* | 1.23 | 1.20 | 1.44 | 0.546 | 0.952 |
| o__*Clostridiales*; f_; g_ | 2.26 | 3.00 | 0.87 | 0.833 | 0.232 |
| g__*Roseburia* | 0.16 | 0.15 | 0.21 | 0.060 | 0.792 |
| g__*Blautia* | 1.13 | 1.03 | 1.53 | 0.437 | 0.724 |
| g__*Klebsiella* | 0.01 | 0.08 | 0.01 | 0.028 | 0.109 |
| g__*Megasphaera* | 2.02 | 1.99 | 2.66 | 0.717 | 0.774 |
| g__*Anaerovibrio* | 0.89 | 1.64 | 3.24 | 1.682 | 0.614 |
| f__*Christensenellaceae*; g_ | 2.92 | 2.36 | 3.38 | 1.322 | 0.873 |
| o__*Bacteroidales*; f_; g_ | 1.04 | 2.15 | 1.26 | 0.744 | 0.532 |
| g__*Coprococcus* | 0.11 | 0.16 | 0.10 | 0.050 | 0.683 |
| g__[*Prevotella*] | 0.22 | 0.83 | 0.60 | 0.248 | 0.205 |
| g__[*Ruminococcus*] | 2.42 | 1.39 | 1.82 | 0.462 | 0.273 |
| g__[*Eubacterium*] | 1.51 | 0.87 | 0.84 | 0.375 | 0.340 |
| g__*Dorea* | 0.75 | 0.43 | 0.44 | 0.149 | 0.207 |
| g__*Treponema* | 0.43 | 2.14 | 1.75 | 0.851 | 0.309 |
| g__*Streptococcus* | 0.64 | 0.58 | 0.94 | 0.365 | 0.783 |
| g__*Faecalibacterium* | 0.47 | 0.23 | 0.38 | 0.182 | 0.622 |
| g__*Succinivibrio* | 0.29 | 0.46 | 0.31 | 0.187 | 0.779 |
| g__*Veillonella* | 0.19 | 0.31 | 0.23 | 0.090 | 0.634 |
| f__[*Mogibacteriaceae*]; g_ | 1.30 | 0.68 | 0.88 | 0.517 | 0.667 |
| g__*Butyricimonas* | 0.58 | 0.67 | 0.62 | 0.190 | 0.935 |
| g__*Collinsella* | 0.49 | 0.78 | 0.63 | 0.150 | 0.367 |
| g__*Turicibacter* | 0.38 | 0.66 | 0.41 | 0.266 | 0.718 |
| g__*Campylobacter* | 0.31 | 0.52 | 1.08 | 0.311 | 0.216 |
| g__*Clostridium* | 0.95 | 1.66 | 0.46 | 0.594 | 0.392 |
| f__*Coriobacteriaceae*; g_ | 1.03 | 0.48 | 0.32 | 0.449 | 0.490 |
| g__*Pasteurella* | 0.01 | 0.03 | 0.06 | 0.014 | 0.106 |
| g__*Sutterella* | 0.32 | 0.45 | 0.24 | 0.157 | 0.653 |
| f__*Rikenellaceae*; g_ | 1.56 | 0.85 | 0.69 | 0.304 | 0.102 |
| g__*Mitsuokella* | 0.45 | 0.01 | 0.20 | 0.302 | 0.568 |
| g__*Actinobacillus* | 0.08 | 0.15 | 0.12 | 0.061 | 0.719 |
| f__*RFP12*; g_ | 0.01 | 0.19 | 0.03 | 0.060 | 0.085 |
| g__*Desulfovibrio* | 0.73 | 0.67 | 0.87 | 0.134 | 0.590 |
| g__*Butyricicoccus* | 0.04 | 0.11 | 0.06 | 0.025 | 0.117 |
| g__*Parabacteroides* | 0.99 | 0.14 | 0.27 | 0.515 | 0.429 |
| g__Corynebacterium | 0.08 | 0.13 | 0.04 | 0.039 | 0.321 |
| g__*p-75-a5* | 0.33 | 0.40 | 0.29 | 0.196 | 0.932 |
| g__*Bulleidia* | 0.39 | 0.15 | 0.54 | 0.206 | 0.433 |
| f__*Peptostreptococcaceae*; g_ | 0.07 | 0.12 | 0.15 | 0.061 | 0.691 |
| g__*Peptostreptococcus* | 0.02 | 0.03 | 0 | 0.011 | 0.261 |
| g__*Bifidobacterium* | 0.14 | 0.12 | 0.09 | 0.058 | 0.819 |
| g__*Acidaminococcus* | 0.85 | 1.15 | 0.74 | 0.763 | 0.932 |
| f__*RF16*; g_ | 0.01 | 0 | 0.05 | 0.016 | 0.103 |
| f__*Prevotellaceae*; g_ | 0.44 | 0.59 | 0.93 | 0.296 | 0.498 |
| f__*Pirellulaceae*; g_ | 0.16 | 0.52 | 0.60 | 0.185 | 0.209 |
| g__Synergistes | 2.59 | 0.46 | 1.06 | 0.935 | 0.235 |
| g__*CF231* | 0.09 | 0.05 | 0.07 | 0.040 | 0.715 |
| Day 31 of life |  |  |  |  |  |
| g__*Fusobacterium* | 0.02 | 0.01 | 0.03 | 0.013 | 0.666 |
| g__*Prevotella* | 11.75 | 11.72 | 8.01 | 1.801 | 0.270 |
| g__*Bacteroides* | 0.37 | 0.28 | 0.24 | 0.141 | 0.824 |
| g__*Lactobacillus* | 2.62 | 2.89 | 3.56 | 0.500 | 0.423 |
| g__*Escherichia* | 0.10 | 0.22 | 0.21 | 0.064 | 0.354 |
| f__*Lachnospiraceae*; g_ | 7.89 | 6.45 | 7.02 | 1.185 | 0.685 |
| f__*Ruminococcaceae*; g_ | 9.43 | 7.16 | 8.32 | 0.916 | 0.220 |
| f__*S24-7*; g_ | 4.92 | 4.99 | 5.52 | 0.736 | 0.830 |
| g__*Oscillospira* | 4.78 | 4.51 | 5.61 | 0.806 | 0.615 |
| g__*Clostridium* | 2.12 | 2.86 | 2.98 | 0.493 | 0.425 |
| f__*Clostridiaceae*; g_ | 5.03 | 5.76 | 5.78 | 0.818 | 0.765 |
| g__*Ruminococcus* | 3.18 | 4.31 | 2.81 | 0.593 | 0.182 |
| g__*Phascolarctobacterium* | 2.73 | 2.69 | 2.34 | 0.322 | 0.655 |
| g__*SMB53* | 3.98 | 4.68 | 4.74 | 0.628 | 0.644 |
| o__*Clostridiales*; f_; g_ | 4.59 | 3.03 | 4.88 | 0.836 | 0.246 |
| g__*Roseburia* | 1.20 | 1.47 | 0.63 | 0.323 | 0.194 |
| g__*Blautia* | 0.76 | 0.89 | 0.90 | 0.227 | 0.892 |
| g__*Megasphaera* | 0.43 | 0.54 | 0.27 | 0.184 | 0.583 |
| g__*Anaerovibrio* | 0.66 | 1.00 | 0.69 | 0.432 | 0.824 |
| f__*Christensenellaceae*; g_ | 2.08 | 3.19 | 5.52 | 0.978 | 0.062 |
| o__*Bacteroidales*; f_; g_ | 2.50 | 3.07 | 2.34 | 0.339 | 0.284 |
| g__*Coprococcus* | 1.90 | 2.07 | 1.68 | 0.639 | 0.911 |
| g__[*Prevotella*] | 1.05 | 1.97 | 1.64 | 0.567 | 0.511 |
| g__[*Ruminococcus*] | 1.47 | 1.50 | 1.73 | 0.485 | 0.922 |
| g__[*Eubacterium*] | 1.02 | 0.94 | 0.74 | 0.233 | 0.703 |
| g__*Dorea* | 1.01 | 0.56 | 0.54 | 0.250 | 0.343 |
| g__*Treponema* | 1.84 | 3.24 | 2.28 | 0.561 | 0.197 |
| g__*Streptococcus* | 0.03 | 0.07 | 0.05 | 0.030 | 0.614 |
| g__*Faecalibacterium* | 0.67 | 0.56 | 0.33 | 0.180 | 0.425 |
| g__*Succinivibrio* | 0.84 | 0.93 | 0.79 | 0.310 | 0.948 |
| g__*Veillonella* | 0.01 | 0.02 | 0.01 | 0.010 | 0.758 |
| f__[*Mogibacteriaceae*]; g_ | 1.23 | 0.91 | 1.29 | 0.146 | 0.144 |
| g__*Butyricimonas* | 0.03 | 0.06 | 0.04 | 0.027 | 0.770 |
| g__*Collinsella* | 1.44 | 0.95 | 1.16 | 0.373 | 0.648 |
| g__*Turicibacter* | 1.25 | 1.78 | 1.94 | 0.349 | 0.360 |
| g__*Campylobacter* | 0.25 | 0.20 | 0.28 | 0.066 | 0.641 |
| g__*Clostridium* | 0.02 | 0 | 0.02 | 0.006 | 0.215 |
| f__*Coriobacteriaceae*; g_ | 0.34 | 0.30 | 0.26 | 0.051 | 0.582 |
| g__*Sutterella* | 0.11 | 0.11 | 0.14 | 0.038 | 0.837 |
| f__*Rikenellaceae*; g_ | 0 | 0.06 | 0.02 | 0.026 | 0.237 |
| g__*Mitsuokella* | 0.22 | 0.09 | 0.03 | 0.074 | 0.226 |
| g__*Sarcina* | 0.42 | 0.40 | 0.18 | 0.117 | 0.302 |
| g__*Actinobacillus* | 0 | 0.02 | 0.01 | 0.010 | 0.632 |
| f__*RFP12*; g_ | 1.61 | 1.26 | 1.11 | 0.337 | 0.581 |
| g__*Desulfovibrio* | 0.23 | 0.31 | 0.29 | 0.045 | 0.441 |
| g__*Butyricicoccus* | 0.42 | 0.27 | 0.32 | 0.069 | 0.336 |
| g__*Parabacteroides* | 0.25 | 0.42 | 0.49 | 0.189 | 0.682 |
| g__*Corynebacterium* | 0.06 | 0.03 | 0.07 | 0.015 | 0.221 |
| g__*p-75-a5* | 0.32 | 0.31 | 0.28 | 0.141 | 0.977 |
| g__*Bulleidia* | 0.11 | 0.12 | 0.20 | 0.043 | 0.263 |
| f__*Peptostreptococcaceae*; g_ | 0.41 | 0.32 | 0.27 | 0.084 | 0.508 |
| g__*Bifidobacterium* | 0.01 | 0.02 | 0.04 | 0.021 | 0.747 |
| g__*Acidaminococcus* | 0.05 | 0.17 | 0.11 | 0.063 | 0.398 |
| f__*RF16*; g_ | 0.67 | 0.67 | 0.33 | 0.181 | 0.339 |
| f__*Prevotellaceae*; g_ | 0.58 | 0.50 | 0.50 | 0.234 | 0.960 |
| f__*Pirellulaceae*; g_ | 0.85 | 1.20 | 0.59 | 0.236 | 0.197 |
| g__*Synergistes* | 0.01 | 0.04 | 0.03 | 0.019 | 0.629 |
| g__*CF231* | 0.36 | 0.35 | 0.35 | 0.075 | 0.994 |
| Day 34 of life |  |  |  |  |  |
| g__*Prevotella* | 22.39 | 22.23 | 24.89 | 2.108 | 0.625 |
| g__*Bacteroides* | 0.05 | 0.05 | 0.02 | 0.019 | 0.471 |
| g__*Lactobacillus* | 5.48 | 4.77 | 5.90 | 0.783 | 0.590 |
| g__*Escherichia* | 0.47 | 0.20 | 0.11 | 0.161 | 0.281 |
| f__*Lachnospiraceae*; g_ | 5.94 | 4.69 | 5.56 | 0.811 | 0.524 |
| f__*Ruminococcaceae*; g_ | 7.90 | 6.02 | 5.84 | 0.763 | 0.125 |
| f__*S24-7*; g_ | 3.08 | 3.30 | 3.45 | 0.403 | 0.814 |
| g__*Oscillospira* | 2.86 | 2.55 | 2.34 | 0.333 | 0.551 |
| g__*Clostridium* | 1.15^b^ | 2.18^a^ | 1.17^b^ | 0.240 | **0.005** |
| f__*Clostridiaceae*; g_ | 3.34^b^ | 6.88^a^ | 3.87^b^ | 0.687 | **0.002** |
| g__*Ruminococcus* | 2.54 | 2.06 | 2.13 | 0.287 | 0.442 |
| g__*Phascolarctobacterium* | 3.35 | 2.55 | 2.74 | 0.288 | 0.132 |
| g__*SMB53* | 2.05^b^ | 4.05^a^ | 2.30^b^ | 0.531 | **0.021** |
| o__*Clostridiales*; f_; g_ | 1.82 | 1.72 | 1.75 | 0.405 | 0.983 |
| g__*Roseburia* | 3.08^b^ | 4.79^ab^ | 6.10^a^ | 0.810 | ^0.049^ |
| g__*Blautia* | 2.60 | 3.06 | 2.79 | 0.579 | 0.846 |
| g__*Megasphaera* | 2.89 | 1.26 | 2.84 | 0.696 | 0.170 |
| g__*Anaerovibrio* | 1.30 | 1.72 | 2.59 | 0.396 | 0.089 |
| f__*Christensenellaceae*; g_ | 0.39 | 0.46 | 0.38 | 0.182 | 0.942 |
| o__*Bacteroidales*; f_; g_ | 1.50 | 1.65 | 1.42 | 0.246 | 0.809 |
| g__*Coprococcus* | 2.24 | 2.94 | 2.44 | 0.304 | 0.247 |
| g__[*Prevotella*] | 1.28 | 1.23 | 1.81 | 0.243 | 0.207 |
| g__[*Ruminococcus*] | 0.63 | 0.37 | 0.31 | 0.117 | 0.138 |
| g__[*Eubacterium*] | 1.11 | 0.63 | 0.97 | 0.158 | 0.091 |
| g__*Dorea* | 1.35 | 0.72 | 0.67 | 0.251 | 0.123 |
| g__*Treponema* | 1.40 | 1.20 | 0.57 | 0.361 | 0.275 |
| g__*Streptococcus* | 0.38 | 0.33 | 0.40 | 0.122 | 0.920 |
| g__*Faecalibacterium* | 1.76 | 1.66 | 2.05 | 0.351 | 0.725 |
| g__*Succinivibrio* | 1.18 | 1.20 | 0.67 | 0.533 | 0.746 |
| f__[*Mogibacteriaceae*]; g_ | 0.61 | 0.53 | 0.36 | 0.095 | 0.199 |
| g__*Collinsella* | 0.71 | 0.78 | 0.60 | 0.179 | 0.772 |
| g__*Turicibacter* | 0.31^b^ | 0.91^a^ | 0.32^b^ | 0.143 | **0.006** |
| g__*Campylobacter* | 0.21 | 0.07 | 0.13 | 0.064 | 0.288 |
| g__*Clostridium* | 0.01 | 0.01 | 0.01 | 0.005 | 0.398 |
| f__*Coriobacteriaceae*; g_ | 0.51 | 0.45 | 0.42 | 0.059 | 0.539 |
| g__*Sutterella* | 0.08 | 0.07 | 0.07 | 0.016 | 0.934 |
| g__*Mitsuokella* | 0.83 | 0.56 | 1.02 | 0.205 | 0.298 |
| g__*Sarcina* | 1.31 | 1.12 | 1.20 | 0.223 | 0.835 |
| f__*RFP12*; g_ | 0.76 | 0.61 | 0.27 | 0.217 | 0.307 |
| g__*Desulfovibrio* | 0.14 | 0.11 | 0.13 | 0.022 | 0.619 |
| g__*Butyricicoccus* | 0.63 | 0.56 | 0.48 | 0.085 | 0.469 |
| g__*Parabacteroides* | 0.25 | 0.13 | 0.11 | 0.076 | 0.385 |
| g__*Corynebacterium* | 0.04 | 0.05 | 0.02 | 0.011 | 0.243 |
| g__*p-75-a5* | 0.13 | 0.21 | 0.13 | 0.056 | 0.522 |
| g__*Bulleidia* | 0.46 | 0.35 | 0.49 | 0.082 | 0.451 |
| f__*Peptostreptococcaceae*; g_ | 0.49 | 0.55 | 0.60 | 0.072 | 0.585 |
| g__*Bifidobacterium* | 0.06 | 0.38 | 0.10 | 0.103 | 0.065 |
| g__*Acidaminococcus* | 0.16 | 0.11 | 0.18 | 0.055 | 0.659 |
| f__*RF16*; g_ | 1.10^a^ | 0.38^b^ | 0.18^b^ | 0.262 | **0.048** |
| f__*Prevotellaceae*; g_ | 0.06 | 0.07 | 0.03 | 0.025 | 0.484 |
| f__*Pirellulaceae*; g_ | 0.14 | 0.26 | 0.08 | 0.090 | 0.376 |
| g__*CF231* | 0.41 | 0.32 | 0.37 | 0.052 | 0.455 |
| Day 38 of life |  |  |  |  |  |
| g__*Prevotella* | 26.57 | 23.26 | 26.08 | 1.274 | 0.143 |
| g__*Bacteroides* | 0.02 | 0.02 | 0.01 | 0.010 | 0.500 |
| g__*Lactobacillus* | 5.53 | 6.61 | 5.70 | 1.213 | 0.787 |
| g__*Escherichia* | 0.10 | 0.19 | 0.24 | 0.065 | 0.342 |
| f__*Lachnospiraceae*; g_ | 6.00 | 5.23 | 5.52 | 0.956 | 0.845 |
| f__*Ruminococcaceae*; g_ | 5.47^b^ | 6.72^a^ | 5.24^b^ | 0.428 | **0.040** |
| f__*S24-7*; g_ | 3.13 | 3.73 | 3.07 | 0.388 | 0.408 |
| g__*Oscillospira* | 1.98 | 2.19 | 1.73 | 0.258 | 0.463 |
| g__*Clostridium* | 1.10 | 1.18 | 1.17 | 0.262 | 0.972 |
| f__*Clostridiaceae*; g_ | 3.54 | 5.01 | 5.08 | 0.850 | 0.363 |
| g__*Ruminococcus* | 1.57 | 1.64 | 1.58 | 0.212 | 0.966 |
| g__*Phascolarctobacterium* | 2.66 | 2.72 | 2.89 | 0.206 | 0.715 |
| g__*SMB53* | 1.96 | 2.79 | 3.15 | 0.491 | 0.239 |
| o__*Clostridiales*; f_; g_ | 1.45 | 1.73 | 1.53 | 0.175 | 0.503 |
| g__*Roseburia* | 4.34 | 4.37 | 4.69 | 0.541 | 0.887 |
| g__*Blautia* | 3.62 | 3.13 | 4.36 | 0.514 | 0.257 |
| g__*Megasphaera* | 2.90 | 2.11 | 1.63 | 0.676 | 0.432 |
| g__*Anaerovibrio* | 1.76 | 1.93 | 1.92 | 0.349 | 0.926 |
| f__*Christensenellaceae*; g_ | 0.13 | 0.24 | 0.06 | 0.085 | 0.300 |
| o__*Bacteroidales*; f_; g_ | 1.42 | 1.79 | 1.34 | 0.219 | 0.313 |
| g__*Coprococcus* | 2.48 | 2.90 | 2.58 | 0.221 | 0.370 |
| g__[*Prevotella*] | 1.62 | 1.86 | 2.18 | 0.240 | 0.286 |
| g__[*Ruminococcus*] | 0.76 | 0.59 | 0.51 | 0.121 | 0.340 |
| g__[*Eubacterium*] | 0.77 | 0.62 | 0.73 | 0.115 | 0.633 |
| g__*Dorea* | 1.59 | 0.68 | 0.98 | 0.266 | 0.055 |
| g__*Treponema* | 0.77 | 0.73 | 0.64 | 0.203 | 0.909 |
| g__*Streptococcus* | 0.33 | 0.18 | 0.44 | 0.097 | 0.184 |
| g__*Faecalibacterium* | 1.97 | 1.51 | 1.93 | 0.268 | 0.396 |
| g__*Succinivibrio* | 1.64 | 0.96 | 1.21 | 0.386 | 0.441 |
| f__[*Mogibacteriaceae*]; g_ | 0.35 | 0.47 | 0.24 | 0.081 | 0.137 |
| g__*Collinsella* | 0.43 | 0.40 | 0.41 | 0.085 | 0.952 |
| g__*Turicibacter* | 0.28 | 0.47 | 0.48 | 0.102 | 0.311 |
| g__*Campylobacter* | 0.11 | 0.24 | 0.13 | 0.073 | 0.405 |
| f__*Coriobacteriaceae*; g_ | 0.54 | 0.52 | 0.43 | 0.056 | 0.367 |
| g__*Sutterella* | 0.16 | 0.13 | 0.15 | 0.021 | 0.730 |
| g__*Mitsuokella* | 1.10 | 0.77 | 0.81 | 0.192 | 0.408 |
| g__*Sarcina* | 1.01 | 0.83 | 1.04 | 0.217 | 0.753 |
| f__*RFP12*; g_ | 0.18 | 0.41 | 0.11 | 0.150 | 0.350 |
| g__*Desulfovibrio* | 0.11 | 0.11 | 0.12 | 0.024 | 0.864 |
| g__*Butyricicoccus* | 0.56 | 0.66 | 0.62 | 0.063 | 0.480 |
| g__*Parabacteroides* | 0.23 | 0.18 | 0.14 | 0.064 | 0.638 |
| g__*p-75-a5* | 0.11 | 0.20 | 0.16 | 0.027 | 0.074 |
| g__*Bulleidia* | 0.53 | 0.51 | 0.50 | 0.096 | 0.971 |
| f__*Peptostreptococcaceae*; g_ | 0.61 | 0.70 | 0.71 | 0.071 | 0.562 |
| g__*Bifidobacterium* | 0.35 | 0.82 | 0.67 | 0.191 | 0.213 |
| g__*Acidaminococcus* | 0.26 | 0.10 | 0.13 | 0.052 | 0.077 |
| f__*RF16*; g_ | 0.80 | 0.38 | 0.43 | 0.221 | 0.346 |
| f__*Prevotellaceae*; g_ | 0.01 | 0.03 | 0 | 0.008 | 0.125 |
| f__*Pirellulaceae*; g_ | 0.04 | 0.17 | 0.02 | 0.049 | 0.077 |
| g__*CF231* | 0.41 | 0.56 | 0.43 | 0.086 | 0.418 |
